# Evaluating Adaptive Divergence Between Migratory and Nonmigratory Ecotypes of a Salmonid Fish, *Oncorhynchus mykiss*

**DOI:** 10.1534/g3.113.006817

**Published:** 2013-08-01

**Authors:** Matthew C. Hale, Frank P. Thrower, Ewann A. Berntson, Michael R. Miller, Krista M. Nichols

**Affiliations:** * Department of Biological Sciences, Purdue University, West Lafayette, Indiana 98026; † National Oceanic and Atmospheric Administration, National Marine Fisheries Service, Alaska Fisheries Science Center, Ted Stevens Marine Institute, Juneau, Alaska 99801; ‡ National Oceanic and Atmospheric Administration, National Marine Fisheries Service, Northwest Fisheries Science Center, Seattle, Washington 98112; § Department of Animal Science, University of California, Davis, California 95616

**Keywords:** smoltification, life history variation, genomics, SNP, salmonids

## Abstract

Next-generation sequencing and the application of population genomic and association approaches have made it possible to detect selection and unravel the genetic basis to variable phenotypic traits. The use of these two approaches in parallel is especially attractive in nonmodel organisms that lack a sequenced and annotated genome, but only works well when population structure is not confounded with the phenotype of interest. Herein, we use population genomics in a nonmodel fish species, rainbow trout (*Oncorhynchus mykiss*), to better understand adaptive divergence between migratory and nonmigratory ecotypes and to further our understanding about the genetic basis of migration. Restriction site-associated DNA (RAD) tag sequencing was used to identify single-nucleotide polymorphisms (SNPs) in migrant and resident *O. mykiss* from two systems, one in Alaska and the other in Oregon. A total of 7920 and 6755 SNPs met filtering criteria in the Alaska and Oregon data sets, respectively. Population genetic tests determined that 1423 SNPs were candidates for selection when loci were compared between resident and migrant samples. Previous linkage mapping studies that used RAD DNA tag SNPs were available to determine the position of 1990 markers. Several significant SNPs are located in genome regions that contain quantitative trait loci for migratory-related traits, reinforcing the importance of these regions in the genetic basis of migration/residency. Annotation of genome regions linked to significant SNPs revealed genes involved in processes known to be important in migration (such as osmoregulatory function). This study adds to our growing knowledge on adaptive divergence between migratory and nonmigratory ecotypes of this species; across studies, this complex trait appears to be controlled by many loci of small effect, with some in common, but many loci not shared between populations studied.

Migration, the long-distance movement of organisms to take advantage of seasonal resources, has long interested both scientists and lay people alike. The cues that initiate the cascade of physiological, behavioral, and morphological changes that prepare an individual for migration are varied and complex (Dingle 2006). These cues interact with signals from the environment, such as temperature and food availability, that in concert influence the decision of an individual to migrate (Dingle 2006; Wilcove and Wikelski 2009). Heritability studies across taxonomic groups suggest that both propensity and timing of migration have a genetic component (see Pulido and Berthold 2003 for review), but we have limited insight into the genes and molecular mechanisms involved (Dingle 1991; Liedvogel *et al.* 2011). This paucity of information limits our understanding of the evolution of migration and prevents us from asking such questions as: are the same genetic mechanisms involved in migration conserved across different taxonomic groups? Do the genes that predispose an individual to migrate get inherited as a migratory gene package? And what patterns do we see in genes associated with migration in closely related species that differ in their migratory strategies (Pulido and Berthold 2003; reviewed in Liedvogel *et al.* 2011)?

The salmonid fishes (salmon, trout and charr) are an exemplary taxonomic group to study the genetic basis of migration. Multiple species have both migratory, ocean-going forms and resident individuals that stay in fresh water throughout their life cycle. *Oncorhynchus mykiss* is an especially attractive taxon as it exhibits both life history types, often within the same river. Previous studies in controlled breeding designs have identified quantitative trait loci (QTL) for migration-related traits in this species (Nichols *et al.* 2008; Hecht *et al.* 2012; Le Bras *et al.* 2011) and other salmonids (Norman *et al.* 2011) confirming a genetic basis to migratory related traits. In addition, genome-wide association approaches (GWAS) have been used and, like QTL studies, have pointed to many different regions of the genome being associated with migration (Hecht *et al.* 2013). However, both approaches have their limitations, especially when applied in natural systems. For example, QTL studies are limited to surveying genotype-phenotype variation in progeny derived from a very small subset of individuals sampled within or between population(s). Such sampling captures a limited amount of the total genetic variation that underlies the trait(s) of interest. Meanwhile, association analysis approaches suffer from high false-positive rates due to underlying population structure and/or kin relatedness (*e.g.*, Price *et al.* 2006; Ingvarsson and Street 2011). These false-positive results can be corrected by accounting for population structure; however, such methods can both induce false-negative results (where true small effect associations are removed) where population structure is highly correlated with phenotype. The latter problem may be of special concern in salmonids because resident populations can be land locked by physical barriers that restrict gene flow with neighboring migratory populations (Limborg *et al.* 2012). Population genomic methods offer an alternative approach in the identification of genes that have adaptive significance. The basic premise of such methods is that allele frequencies at selected sites will differ from neutral sites. Recently such methods have been used to highlight regions of the genome under selection for specific traits (Dalziel *et al.* 2009; Nielsen *et al.* 2009; Hohenlohe *et al.* 2010). For example, genomic scans in marine and freshwater three-spined sticklebacks (*Gasterosteus aculeatus*) both confirmed, and discovered new loci showing signatures of selection between the two phenotypes (Hohenlohe *et al.* 2010). Popular approaches to finding loci under selection can be broadly split into “empirical” and “theoretical” approaches. Examples of empirical approaches include *F*_ST_ outlier methods that aim to locate outlier loci among many neutral loci (Beaumont and Nichols 1996; Luikart *et al.* 2003), whereas examples of theoretical tests include statistics such as Tajima’s *D* (*e.g.*, Nielsen 2005; Nielsen *et al.* 2007) that test for departures from neutrality.

The advent of genotype-by-sequencing methods has made it possible to identify a large number of polymorphisms throughout a genome at a relatively low cost, and in doing so, has made genome-wide population genomic and association tests possible in nonmodel organisms (Baird *et al.* 2008; Miller *et al.* 2007; references in Seeb *et al.* 2011). The use of restriction site−associated DNA (RAD) tag sequencing is especially attractive because tens of thousands of SNPs can be found relatively quickly and cheaply (reviewed in Rowe *et al.* 2011 and Davey *et al.* 2011). For example, RAD tag sequencing has made it possible to construct linkage maps (Amores *et al.* 2011; Baxter *et al.* 2011), conduct population genomic studies (Hohenlohe *et al.* 2010), perform QTL analyses (Chutimanitsakun *et al.* 2011; Miller *et al.* 2012; Pfender *et al.* 2011; Hecht *et al.* 2012), and perform GWAS analysis (Hecht *et al.* 2013) in organisms that previously did not have extensive SNP resources.

In this study, we used Illumina RAD tag methods in two populations of *O. mykiss* to identify genetic loci associated with migration and residency using population genomic approaches to question whether the same markers are divergent between migratory and resident ecotypes in each geographic location (*i.e.*, common mechanisms). In the absence of a fully sequenced and annotated genome, this study illustrates the ability to conduct whole-genome studies on an ecologically and evolutionarily important trait with the aid of high-throughput genomic sequencing.

## Materials and Methods

### Sampling and DNA extraction

Resident and migratory individuals were sampled from each of two populations: Sashin Creek, Alaska and Little Sheep Creek, Oregon. Hereafter, the migrant steelhead trout form of *O. mykiss* will be referred to as migrant trout and the resident rainbow trout form as resident trout. Upon returning from the ocean to their natal streams to spawn, all migrants were sampled at sexual maturity. Resident trout from the Sashin Creek, Alaska population also were sampled at sexual maturity (tested for the expression of gametes). The Sashin Creek residents are separated from returning migrants by two barrier waterfalls. All migrants sampled were downstream of these waterfalls, whereas all residents sampled were upstream (see Thrower *et al.* 2004 for more details). The migrant and resident individuals in the Little Sheep Creek population are not separated by barrier waterfalls (see Berntson *et al.* 2011 for more details) and so samples of both phenotypes were taken from the same area. Resident trout from the Little Sheep Creek, Oregon, population were determined to be resident as determined by size threshold (minimum fork length of 160 mm). Both river catchments have active hatchery programs; to limit effects of selection due to hatchery programs, only wild fish were sampled (upon release, all hatchery fish have the adipose fin removed). At sampling, the fork length (tip of snout to the fork of the caudal fin) and sex of the fish was recorded, and a portion of the caudal fin was removed and stored in ethanol for DNA extraction. The fish were immediately released after processing. DNA was extracted for a total of 386 fish (105 Sashin Creek migrants, 90 Sashin Creek residents, 100 Little Sheep Creek migrants, and 91 Little Sheep Creek residents) using either standard phenol:chloroform procedures (Wasko *et al.* 2003) or QIAGEN DNeasy Tissue Extraction kits (QIAGEN Inc., Valencia, CA). All Sashin Creek residents were sampled in 2008 and 2010, and Sashin Creek migrants were sampled in all years from 2006 to 2010. Little Sheep Creek samples for both migrants and residents were collected in 2011.

### RAD library construction

A total of 500 ng of DNA was used from each sample (n = 386 in total) to construct RAD tag libraries for sequencing as described in Miller *et al.* (2012). To summarize, DNA from each individual was digested with *SbfI* and then barcoded with a unique 6-base sequence 3′ to the *SbfI* cut site. Barcoded DNA from 15 samples was pooled and fragmented using the Sonic Ruptor 400 (Omni International, Kennesaw, GA). Fragments were size selected between 300 and 600 bp, blunt-ended, and an A nucleotide was added to the 3′ end, to which the Illumina P2 sequencing primer was ligated. The library was amplified by polymerase chain reaction (PCR; 96° for 3 min then 14 cycles of 96° for 15 sec, 60° for 15 sec, and 72° for 30 sec followed by 5 min at 72°), and again size selected for fragment sizes 300−600 bp. Libraries were quantified using the KAPA SYBR FAST qPCR kit (KAPA Biosystems, Woburn, MA) on the StepOne Plus (Applied Biosystems, Foster City, CA) real-time PCR platform.

### Bioinformatics

Sequenced libraries were processed as described by Miller *et al.* (2012) using Perl scripts and Novocraft software (scripts are available as supplemental material in Miller *et al.* (2012)). To summarize, reads were trimmed to 89 bp to remove barcodes and the *SbfI* overhang and then quality filtered. Reads were assembled *de novo* to construct a database for identifying SNPs. The SNP database was constructed from 20 individuals: 10 from Sashin Creek (5 migrants and 5 residents) and 10 from Little Sheep Creek (5 migrants and 5 residents). To find loci variable both within and between populations, SNPs were found by identifying polymorphisms in three separate comparisons of these individuals: one constructed from all 20 fish, one from the 10 fish from Little Sheep Creek, and one from the 10 fish from Sashin Creek. Unique alignments of identical sequences with fewer than five reads and more than 200 reads were discarded. Retained alignments were compared for SNPs, which were tentatively separated into two alleles at the same locus. Candidate SNP loci required a minimum depth of coverage of five reads for both alleles. This procedure was repeated for all three databases, and the resulting SNP files for each database were combined to create a reference SNP database, removing redundant loci.

Three genotype datasets were created: (1) all fish from both populations (n = 386 individuals); (2) only fish from Sashin Creek, Alaska (n = 195); and (3) only fish from Little Sheep Creek, Oregon (n = 191). All individuals were genotyped against the database using Bowtie and custom Perl scripts (Miller *et al.* 2012), which report the number of reads for alleles A and B at each SNP. Genotypes were scored only if a minimum total read count of 8 was met. The log_10_ ratio of the number of reads aligning to allele A/ allele B was used to separate homozygotes and heterozygotes. Heterozygotes were determined if the log_10_ ratio was between −0.61 and 0.61 (one SD around the mean) with AA homozygotes scored if the ratio was greater than 0.9 and BB homozygotes if the ratio was less than −0.9 (as in Hecht *et al.* 2012). Reads that fell outside these thresholds were removed.

### SNP filtering

Salmonids have experienced a relatively recent whole-genome duplication (between 25 and 100 million years ago; Allendorf and Thorgaard 1984), which can lead to the erroneous identification of candidate SNPs from the alignment of paralogs that are very nearly identical (Everett *et al.* 2011; Miller *et al.* 2012). Paralogous sequence variants (PSVs) are false SNPs that should be removed from the dataset. As also described by Miller *et al.* (2012), we performed RAD tag sequencing on two doubled haploid *O. mykiss* that should have no heterozygous positions within any one individual genome; any sequence producing a heterozygous genotype in doubled haploid *O. mykiss* is identified as a PSV and were removed. Candidate PSVs have also been shown to produce an excess of heterozygous genotypes (M. Everett, unpublished data; Hohenlohe *et al.* 2011). Therefore, any SNP that produced an observed number of heterozygous genotypes greater than 90% was removed. SNPs were further filtered by minimum minor allele frequency <2%, that were missing >50% genotypes and were out of Hardy Weinberg Equilibrium (−log_10_ *P* = > 5.0) as measured in each dataset. The combined dataset also was filtered to only include markers that were polymorphic in both the Sashin Creek and Little Sheep Creek populations.

### Genomic ordering and annotation of SNPs

To provide information on the genomic location of SNPs in this study, polymorphic RAD tags were aligned to two recent *O. mykiss* linkage maps produced from RAD tag SNPs (Miller *et al.* 2012; Hecht *et al.* 2012) and a draft version of the *O. mykiss* genome, using Bowtie. In both cases, RAD tags were considered matches if they only produced one significant hit (allowing 2 mismatches). The draft version of the *O. mykiss* genome consists of sequenced scaffolds some of which have been linked to the physical contigs sequenced at the Clemson University Genomics Institute center (Palti *et al.* 2011). To annotate the genomic regions linked to the top significant SNPs in this study, each scaffold associated with a filtered RAD tag was annotated using BLASTn (minimum e-value = 1 × 10^−20^, minimum of 85 bp of continuous alignment (tags are 89 bp after quality-filter). Identity was confirmed if the tag only produced a significant hit (e-value < 1.0^−20^, at least 85 bp of continuous alignment) to one scaffold. Scaffolds with significant hits were then annotated using BLASTn against the nt database and BLASTx against the nr database in Genbank, as well as against the zebrafish genome (http://useast.ensembl.org/info/data/ftp/index.html) and a compiled annotated salmonid EST database [downloaded from cGRASP (web.uvic.ca/grasp/) and NAGRP (www.csrees.usda.gov/nea/animals/in_focus/an_breeding_if.nagrp.html); date of download March 27, 2012]. Minimum requirements for annotation were a contiguous alignment of 300 bp, with 85% sequence similarity and a minimum e-value of 1 × 10^−20^. As only approximately one quarter of the SNPs were mapped we will report both the number of mapped SNPs and the total number of SNPs that were significant for any statistical test.

### Population genomic analyses

Individual global and pairwise (between residents and migrants) *F*_ST_ was estimated for each locus in GenAlEx 6.4 (Peakall and Smouse 2006), as was mean expected (H_E_) and observed (H_O_) heterozygosity and the number of private alleles. To identify outlier markers that may be candidates for selection, LOSITAN (Beaumont and Nichols 1996; Antao *et al.* 2008) was used with filtered loci from Sashin Creek and Little Sheep Creek with the following parameters: 55,000 simulations, confidence interval of 0.995, and a false discovery rate of 0.05. Simulated *F*_ST_ was 0.032 for Sashin Creek and 0.001 for Little Sheep Creek, and estimated *F*_ST_ was 0.069 and 0.003 for the two populations, respectively. We used loci with a probability greater than 0.995 to infer candidates for positive selection and neutral loci were defined as having a probability between 0.9 and 0.1.Tajima’s *D* was calculated to estimate the allele frequency spectrum within each of the filtered RAD tags. PoPoolations (version 2.1; Kofler *et al.* 2011) was used to estimate within population Tajima’s *D* by pooling 50 samples from each of the four subpopulations (Sashin Creek and Little Sheep Creek, migrants and residents) and using a window size of 100 base pairs, a minimum rare allele count of 2, a minimum depth of coverage of 4 and a population size estimate of 50, the same parameters were used to estimate nucleotide diversity (Watterson’s theta; Watterson 1975). To generate a smooth, genome-wide distribution of these statistics, a kernel smoothing average using a Gaussian function as described in Hohenlohe *et al.* (2010). This approach was used only on markers with mapping information. Linkage disequilibrium (LD) was calculated as *D*′ (Lewontin 1964) between all pairs of SNPs that were matched to loci in Miller *et al.* (2012). The chromosome and distance between markers was inferred from Miller *et al.* (2012). Tests were run separately for each population, and average *D*′ per chromosome was calculated. A two-tailed *t*-test was used to test the hypothesis that average chromosome-wide *D*′ was different between the two populations. *D*′ calculations were performed in JMP Genomics (SAS Inc., Cary, NC).

## Results

### SNP filtering

Twenty-six lanes of sequencing were conducted using various Illumina instruments (see Supporting Information, File S1). The average number of quality-filtered reads per individual was 3.17 million [± 1.26 million (SEM)]. Illumina reads for each individual (quality filtered and trimmed reads) are deposited at Data Dryad (doi:10.5061/dryad.c6fb2). A total of 30,642 unique candidate biallelic SNPs were identified from 26,459 RAD tags (22,590 tags had a single SNP, 2796 tags had 2 SNPs, and 820 tags had 3 SNPs). Removing SNPs that were heterozygous in either of the sequenced doubled haploids or that showed an observed heterozygosity ≥90% reduced the total number of SNPs to 28,863. Removing SNPs with >50% missing genotypes, minor allele frequency <0.02, and markers out of Hardy-Weinberg equilibrium (and only including markers that were polymorphic in both populations for the combined dataset) further reduced the number of SNPs to 7920, 6755, and 5185 for the Sashin Creek, Little Sheep Creek, and the combined datasets, respectively. RAD tags were compared with Miller *et al.* (2012) and Hecht *et al.* (2012a), and positive matches were named the same with new SNPs following the same nomenclature (see File S2).

### Genomic ordering and annotation of SNPs

A total of 1990 (25% of total) filtered SNPs were matched to two RAD tag−based genetic linkage maps (Miller *et al.* 2012; Hecht *et al.* 2012). This number does not include SNPs that matched multiple tags in Miller *et al.* (2012). In this way, synteny between this study and previous linkage and QTL mapping studies in *O. mykiss* could be inferred from shared microsatellite and SNP markers. In *O. mykiss*, linkage groups have further been matched to physical chromosomes by fluorescence *in situ* hybridization (Phillips *et al.* 2006).

### Population genomics between phenotypes

**Figure 1:**
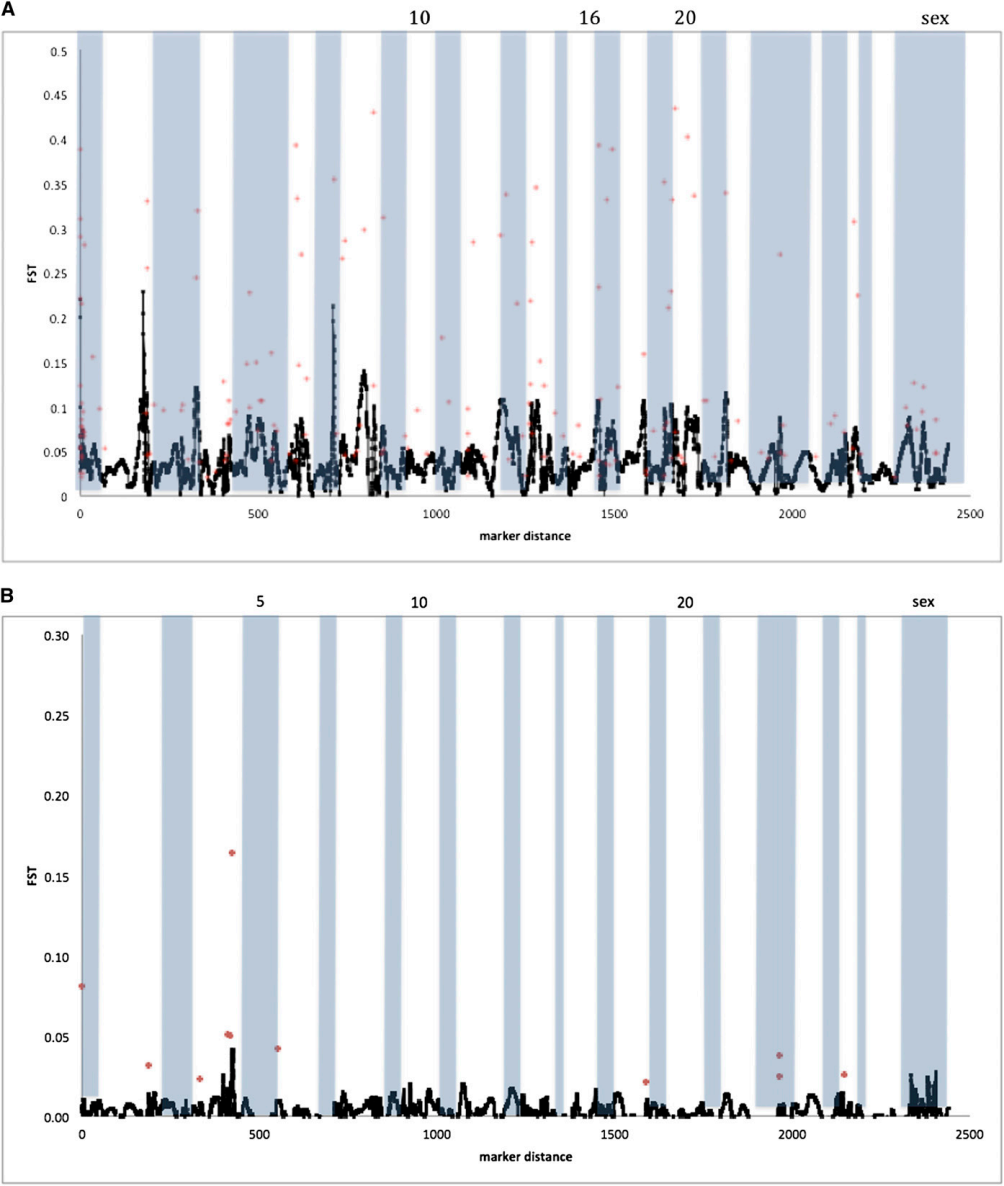
*F*_ST_ for markers that were mapped to two linkage maps of the *O. mykiss* genome (Miller *et al.* 2012; Hecht *et al.* 2012). Markers in red represent significant outliers as determined by LOSITAN (for details, see the section *Materials and Methods*), black line represents the kernel smoothed distribution of all markers (both outliers and “neutral” loci). Chromosomes are marked by alternate shading with a some of the chromosomes labeled. (A) *F*_ST_ between Sashin Creek residents and Sashin Creek migrants and (B) F_ST_ between Little Sheep Creek residents and Little Sheep Creek migrants.

*F*_ST_ between migrants and residents revealed different patterns of variation in the two populations. In Sashin Creek, mean *F*_ST_ between migrants and residents was 0.062 with a range from 0 to 0.459. Mean *F*_ST_ between migrants and residents was much lower in the Little Sheep Creek population (0.003) and ranged from 0 to 0.218. The global comparison between migrants and residents (using both populations) produced a mean *F*_ST_ of 0.075, with values ranging from 0 to 0.510. In *F*_ST_ outlier analyses from LOSITAN, candidates for balancing selection could not be distinguished from loci with an *F*_ST_ of zero and so were not considered further. LOSITAN identified 772, 61, and 55 loci (*P* > 0.995) as candidates for positive selection between residents and migrants in the Sashin Creek, Little Sheep Creek, and global datasets, respectively. Fourteen loci were significant in both the Sashin Creek and Little Sheep Creek samples, and four loci were in common between the global and Sashin Creek datasets. LOSITAN also was used to identify neutral loci, which were defined as those with probabilities between 0.9 and 0.1 (as in Hess *et al.* 2013). We found 5752, 6047, and 3801 loci that fell within this range in the Sashin Creek, Little Sheep Creek, and global populations, respectively. Mapping information was obtained for 178 outlier loci in the Sashin Creek dataset, with at least one significant outlier locus on every chromosome (see Figure 1A). Kernel smoothing showed an increase in the pairwise *F*_ST_ on Omy2, Omy7, and Omy8. A total of 11 loci mapped to eight chromosomes in the Little Sheep Creek analysis of which Omy4 (4) and Omy23 (2) contained multiple outliers. However, kernel smoothing failed to find any chromosomes that seemed to show a significant enrichment for outlier loci (Figure 1B).

**Figure 2:**
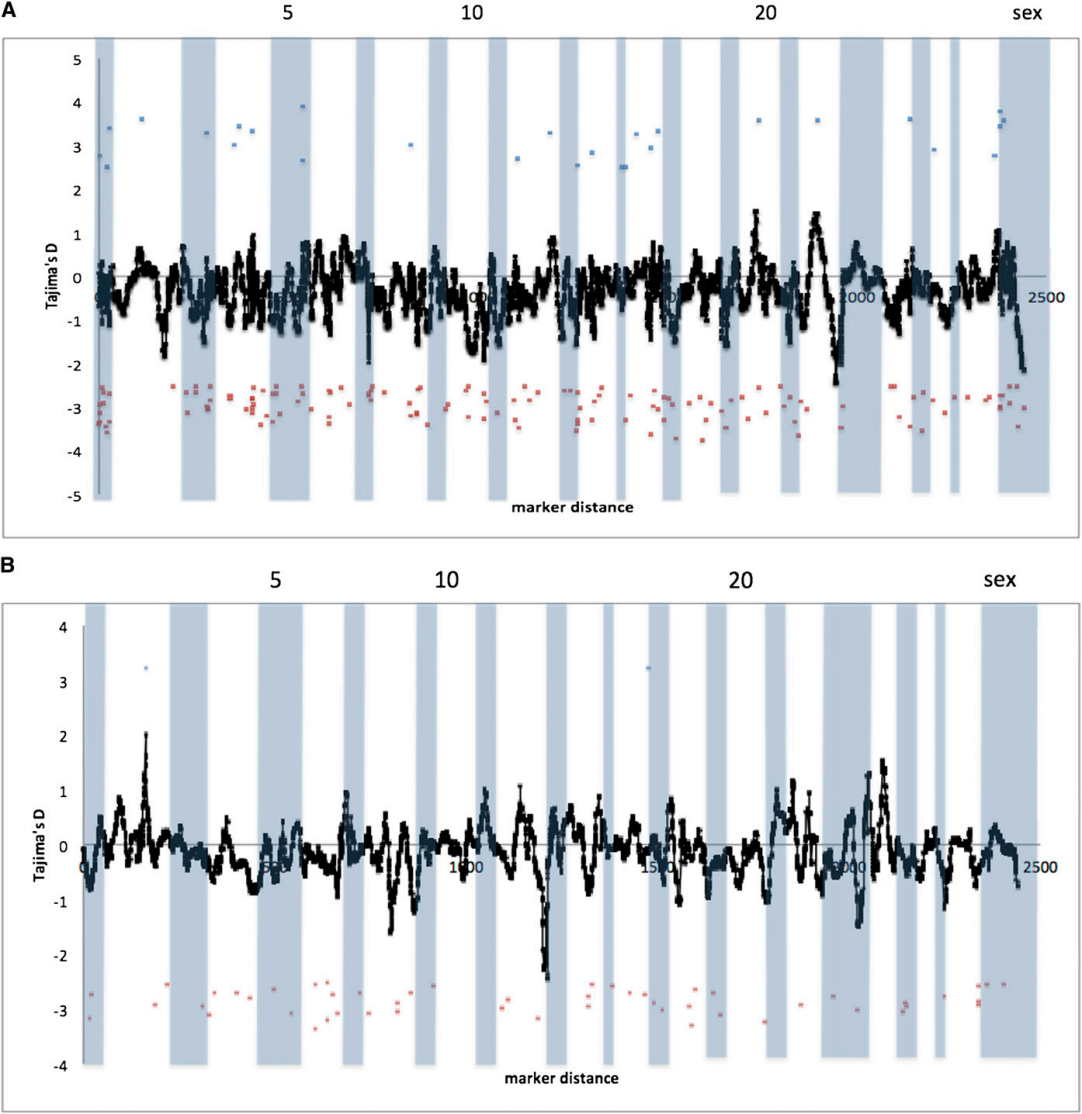
Tajima’s *D* results for markers that mapped to two linkage maps of the *O. mykiss* genome (Miller *et al.* 2012; Hecht *et al.* 2012). Markers in blue produced a significant positive Tajima’s *D* and markers in red a significantly negative Tajima’s *D*. The black line represents the kernel-smoothed average Tajima’s *D* for all mapped markers. Chromosomes are alternately shaded with a subset labeled. (A) Tajima’s *D* calculated within the Sashin migrants, (B) within the Little Sheep Creek samples (combined migrants and residents).

The average Tajima’s *D* statistics for the Sashin Creek migrant population was −0.417 (ranging from −4.003 to 3.902) with 742 loci that differed significantly from neutral expectations. Of these, 109 showed a positive departure from neutrality, or loci that showed an excess of common polymorphisms compared with neutral expectations, whereas 633 loci showed a negative departure from neutrality. These results strongly suggest an excess of low-frequency polymorphisms suggesting population expansion and/or strong effects of purifying selection. Mapping information was obtained for 169 loci that different from neutrality. Of these, 28 had a significant positive value, and 141 had a significantly negative value. Loci with positive Tajima’s *D* values were distributed on 16 chromosomes, whereas all chromosomes had at least one locus with a significant negative *D* value. Kernel smoothing approaches in Sashin Creek migrants suggest Omy2, Omy7, Omy22, and the sex chromosome contain peaks of negative *D* values, whereas Omy20 and Omy22 contain peaks of positive *D* values; note, however, that the magnitude of the positive peaks is smaller than the negative peaks (see Figure 2A). Also note that the majority of the distribution is toward a negative Tajima’s *D*. The Sashin Creek resident population produced an average Tajima’s *D* of −0.003 and ranged from −3.294 to 0.798. Only five loci departed from neutrality, all of which produced a negative *D* value and all of which mapped. The kernel smoothing approach showed the distribution of Tajima’s *D* values was roughly around 0, suggesting neutrality (data not shown) in the resident Sashin Creek population. We combined data from both the migrant and resident individuals within the Little Sheep Creek population as Tajima’s *D* is most powerful at the population level (Tajima 1989) and principle component analysis suggested strongly that both phenotypes form one population (see below). Average Tajima’s *D* was −0.145 with values ranging from −3.36 to 4.13 with 296 markers departing from neutrality (269 negative Tajima’s *D* and 27 positive Tajima’s *D*). Mapping information was obtained for 54 loci with a significant Tajima’s D value. Of these, 52 mapped loci had a significant negative value and two a significant positive value. The negative markers were distributed on every chromosome except Omy13, Omy21, Omy10, Omy24, and Omy26. The two positive loci were on Omy2 and Omy16. Kernel smoothing suggested that Omy12 contained a peak of negative loci with Omy8 and Omy23 smaller peaks. Only Omy2 seemed to show a positive peak (Figure 2B). For details of all significant loci from population genomic analyses see File S3.

**Figure 3:**
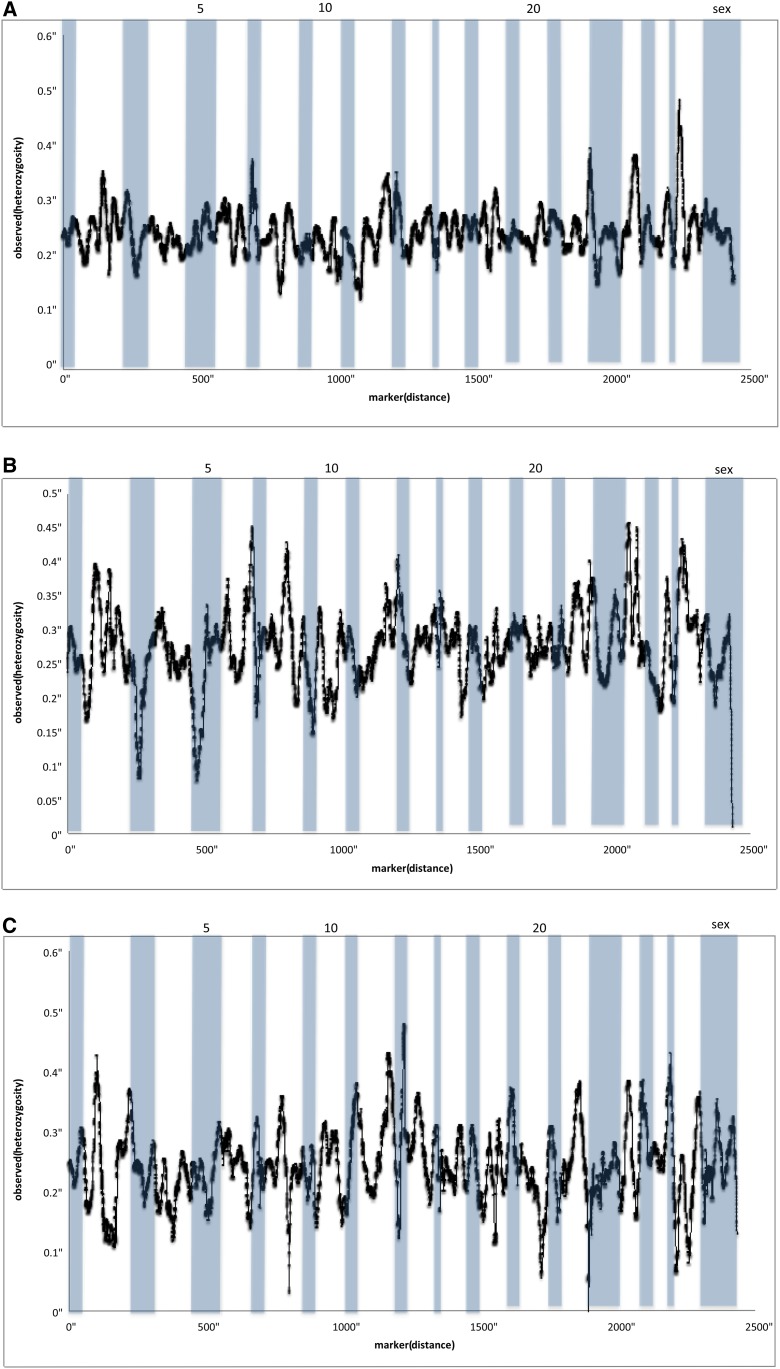
The kernel-smoothed average observed heterozygosity in markers, which mapped to two linkage maps of the *O. mykiss* genome (Miller *et al.* 2012; Hecht *et al.* 2012). The kernel-smoothed average for all markers that mapped is presented for the (A) Sashin migrants, (B) Sashin residents, and (C) Little Sheep Creek samples. Chromosomes are alternately shaded.

**Figure 4:**
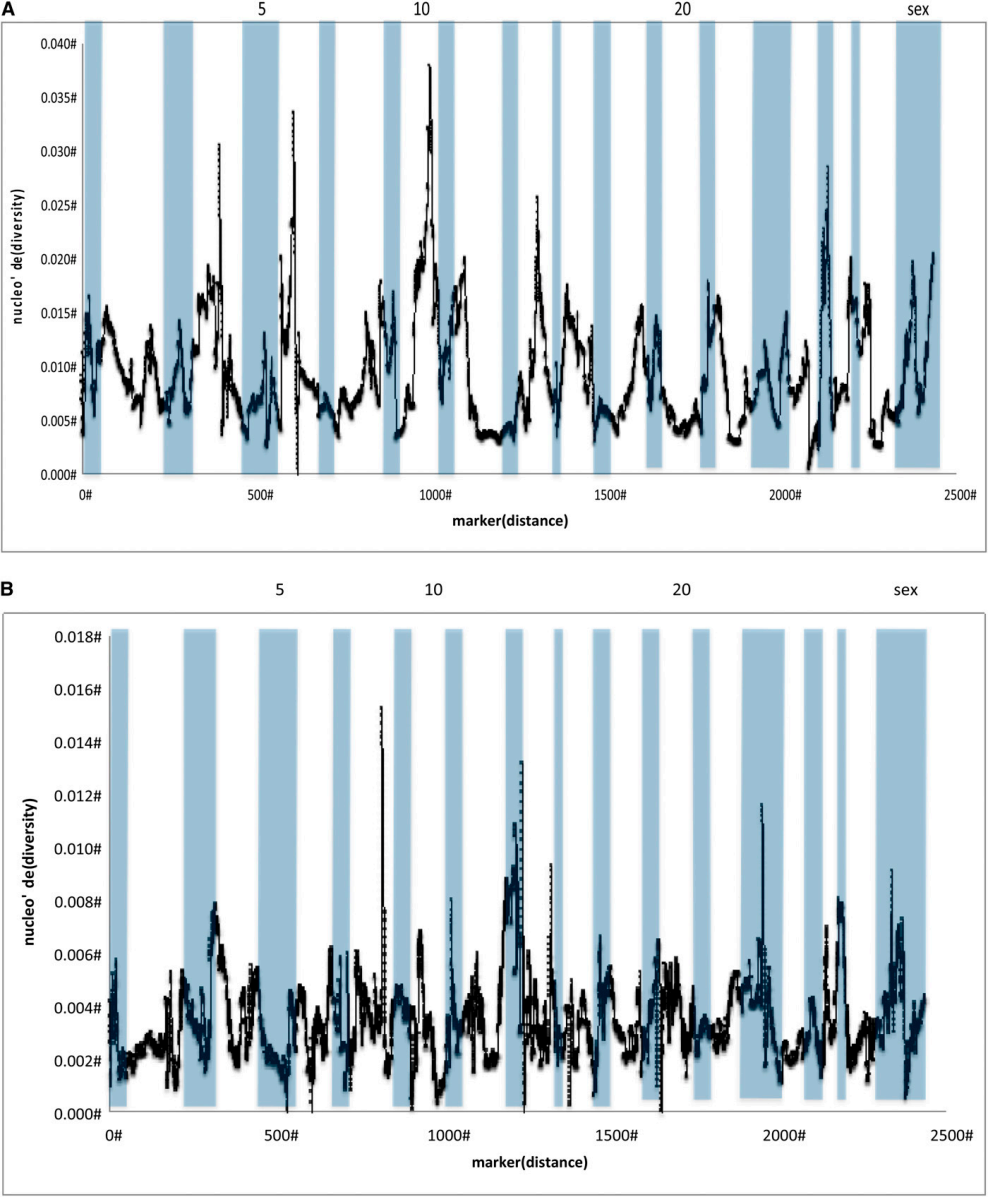
Nucleotide diversity as calculated by Watterson’s theta in markers that mapped to two linkage maps of the *O. mykiss* genome (Miller *et al.* 2012; Hecht *et al.* 2012). The kernel-smoothed average for all markers that mapped is presented for (A) Sashin Creek migrants and (B) Little Sheep Creek samples. Chromosomes are alternately shaded with some chromosomes labeled.

Patterns of observed heterozygosity varied across the genome. The Sashin Creek migrants showed an average observed heterozygosity of 0.229, with peaks of heterozygosity on Omy6, Omy8 and Omy24 and lows on Omy3 and Omy5 (Figure 3A). The Sashin Creek residents had an average observed heterozygosity of 0.246, and appeared to show more variation between chromosomes with peaks of heterozygosity on Omy12, Omy13, and Omy2 and chromosomes with a reduction in heterozygosity on Omy2, Omy8, Omy12, Omy20, and Omy22 (Figure 3B). The Little Sheep Creek samples has an average observed heterozygosity of 0.252 and showed little variation between chromosomes with only Omy27 showing an increase in the observed heterozygosity and no chromosomes showing a large reduction in heterozygosity (see Figure 3C). The Sashin Creek migrants produced an average nucleotide diversity (Watterson’s theta) of 0.008, the Sashin Creek residents 0.0001 and the Little Sheep Creek samples 0.003. Kernel smoothing methods showed that the Sashin Creek migrants have greater-than-average nucleotide diversity on Omy4, Omy6, and Omy10 and reduced levels on Omy2, Omy5, Omy7, Omy12, and Omy24 (Figure 4A). The Sashin Creek residents showed a near 0 nucleotide diversity that did not show much variation between chromosomes (data not shown). The Little Sheep Creek samples showed a generally higher diversity on Omy8, Omy13, and Omy23 and a reduction on Omy10, Omy5 and the sex chromosome (Figure 4B).

We compared the private allele density (alleles that are unique to one population) between the resident and migrant populations in Sashin Creek. A total of 988 loci had private alleles of which 981 had alleles that were unique to the migrant population and 7 loci had alleles that were unique to the resident population. For Tajima’s *D* estimates in the Little Sheep Creek population the migrant and resident phenotypes were combined as they are one population. A total of 52 loci had private alleles of which 23 were unique to the migrant phenotype and 19 were unique to the resident phenotype.

### Linkage disequilibrium

From the 1990 filtered SNPs that were confidently matched to a previous linkage map (Miller *et al.* 2012), average chromosome-wide *D*′ ranged from 0.659 ± 0.013 in the Sashin Creek fish to 0.603 ± 0.009 in the Little Sheep Creek population. The greater LD observed in the Sashin Creek is significantly different than that observed in Little Sheep Creek (*P* < 0.001). Average chromosome-wide *D*′ varied between 0.54 for Omy28 and 0.82 for Omy26 in samples from Sashin Creek, and between 0.49 for Omy21 and 0.68 for Omy25 and Omy22 in Little Sheep Creek fish.

### BLAST analysis

A total of 221 RAD tags that contained both SNPs that produced a significant statistic from population genetic analyses, and mapping information from previous linkage maps (Miller *et al.* 2012; Hecht *et al.* 2012), were BLASTed to scaffolds from a draft version of the *O. mykiss* genome. Of which, 101 RAD tags produced a unique match to one scaffold (e ≤ 1.0^-20^; see File S4). Of these 64 scaffolds produced significant matches to genes with annotation (see Table 1 for results of BLASTn analysis and Table 2 for results of BLASTx analysis).

**Table 1:** BLASTn hits from scaffolds that contain a SNP that produced a significant *F*_ST_ outlier or significant departure from neutrality in the Tajima’s D statistic

SNP ID	Chr	Scaffold	BLASTn Hit	e-Value
R22186	1	MMSRT009C_scaff_1734_1	LDA gene for MHC class I antigen, allele:	0
R33240	1	MMSRT018H_scaff_1292_1	GH2 growth hormone 2 gene	0
R10206	1	MMSRT043A_scaff_1632_1	Cytochrome P450 family 1 subfamily B polypeptide 1 (cyp1b1)	0
R51388	1	MMSRT138E_scaff_1568_1	Cyclin D1 (ccnd1)	0
R34273	2	MMSRT001A_scaff_1294_1	Malate dehydrogenase	1E-90
R41088	3	MMSRT092A_scaff_2616_1	CD83 (Onmy-CD83) gene	0
R29125	5	MMSRT037B_scaff_1504_1	Olfactory receptor family C subfamily 4 member 5 gene	0
R49596	5	MMSRT081B_scaff_1813_1	Dax-1 (Dax1) gene, complete cds	0
R08400	7	MMSRT085C_scaff_1375_1	Alpha-globin and beta-globin, clone 3	0
R19618	7	MMSRT095B_scaff_2418_3	(ADP-ribose) polymerase family	0
R00563	7	MMSRT119G_scaff_1110_1	Adiponectin receptor protein 1 (adr1	0
R00141	9	MMSRT005H_scaff_1052_1	MHC class I a region	0
R13477	9	MMSRT043B_scaff_2167_1	MHC class I antigen (Sasa-UDA) gene	0
R17494	9	MMSRT043G_scaff_1780_1	Dnaj homolog subfamily C member 5	0
R15095	11	MMSRT043C_scaff_1956_1	Myosin regulatory light chain 2	3E-129
R21238	12	MMSRT087F_scaff_1654_1	S toll-like receptor 8a2 (TLR8a2)	0
R35677	12	MMSRT107C_scaff_1855_1	ADP-ribosylation factor 1	0
R20262	13	MMSRT013E_scaff_1784_1	Immunoglobulin heavy chain (igd-A) gene	0
R19667	14	MMSRT001A_scaff_1328_1	Delta-6 fatty acyl desaturase d6fad_a gene	0
R40426	14	MMSRT056B_scaff_1710_1	MHC class I a region	0
R50712	14	MMSRT084F_scaff_1265_2	Tapasin-B (TAPBP)	0
R07628	17	MMSRT033D_scaff_1722_1	Inhibitor of differentiation 1C (ID1C) gene	0
R14298	17	MMSRT108H_scaff_1570_1	GH1 interferon alpha 1-like gene	0
R50966	18	MMSRT045C_scaff_1546_1	Myostatin 2b (MSTN2)	0
R32416	18	MMSRT049G_scaff_1774_1	Na,K-atpase alpha subunit isoform 1b/i (ATP1A1B/i)	0
R28876	19	MMSRT009E_scaff_1603_1	Steroidogenic acute regulatory protein (star) gene	0
R30814	20	MMSRT031B_scaff_1485_1	GH1 interferon alpha 1-like gene	0
R30448	20	MMSRT098B_scaff_1661_1	Gonadotropin subunit beta-2 (gthb2)	0
R27417	21	MMSRT083G_scaff_1866_1	Chaperonin gene, complete cds	0
R40451	23	MMSRT001B_scaff_1711_1	Homeobox protein hoxd13aa (hoxd13aa)	0
R00446	23	MMSRT058G_scaff_1786_1	Growth hormone 2 gene, complete cds	0
R51852	25	MMSRT097D_scaff_1534_1	Potassium-transporting atpase subunit beta-m (at1b4)	0
R04223	sex	MMSRT014E_scaff_2046_1	Carbonyl reductase/20beta-hydroxysteroid dehydrogenase B	0
R37330	sex	MMSRT118G_scaff_1390_1	Polyunsaturated fatty acid elongase (elvol5a) gene	0

**Table 2:** BLASTx hits for scaffolds that contain SNPs that produced an outlier locus in *F*_ST_ analysis or a significant departure from neutrality

SNP ID	Chr	Scaff	GB	Hit	e-Value
R24286	1	MMSRT043A_scaff_1632_1	NP_001167620.1	Cytochrome P450, family 1, subfamily B, polypeptide 1	0
R39129	2	MMSRT081F_scaff_1690_1	ACQ58433.1	C-C chemokine receptor type 9	1E-123
R44099	5	MMSRT068D_scaff_2079_1	NP_001167113.1	Sulfate transporter	0
R27510	5	MMSRT074G_scaff_1637_1	NP_001104690.1	Growth arrest-specific 1a precursor	9E-79
R41103	5	MMSRT133E_scaff_1870_1	NP_001177233.1	Hemicentin-1	4E-68
R10656	8	MMSRT111H_scaff_1511_1	NP_001007455.1	Fucosyltransferase 9	3E-146
R00710	9	MMSRT030E_scaff_2197_1	ACN10547.1	Leucine-rich repeat-containing protein 8D	0
R35771	10	MMSRT055B_scaff_1860_1	XP_003455229.1	Alpha-2 adrenergic receptor	0
R18859	10	MMSRT122H_scaff_1570_1	ACO09612.1	ATP-sensitive inward rectifier potassium channel 15	1E-163
R08544	11	MMSRT057C_scaff_1604_1	XP_003451939.1	Olfactory receptor 51B2	4E-84
R03392	12	MMSRT097H_scaff_1700_1	ABC43375.1	Odorant receptor	1E-73
R19667	14	MMSRT001A_scaff_1328_1	XP_001920036.2	Netrin-1-like	1E-82
R28018	15	MMSRT079E_scaff_1499_1	XP_003448843.1	Potassium voltage-gated channel subfamily D	0
R15666	16	MMSRT106A_scaff_1950_1	XP_003977854.1	Olfactomedin-like protein 3B	4E-94
R05928	17	MMSRT061F_scaff_1802_1	XP_004070492.1	Neurogenic differentiation factor 4	3E-63
R24186	17	MMSRT125C_scaff_1898_1	CBN80811.1	Potassium voltage-gated channel	0
R03565	18	MMSRT049G_scaff_1774_1	ADD60471.1	Na^+^/K+ atpase alpha	6E-135
R41273	18	MMSRT141G_scaff_1929_1	NP_001018411.1	Fidgetin	5E-146
R01482	19	MMSRT050F_scaff_1759_1	ACS35075.1	Thrombospondin-1a	1E-117
R08761	19	MMSRT062C_scaff_2490_1	XP_004084735.1	Metabotropic glutamate receptor 1	5E-109
R03286	20	MMSRT124H_scaff_1869_1	XP_003404464.1	Dystonin-like isoform 2	2E-148
R40464	23	MMSRT038D_scaff_2090_1	NP_571024.1	Forkhead box protein A2	1E-153
R00544	25	MMSRT062C_scaff_2440_1	XP_003448092.1	Protocadherin-18	0
R14689	25	MMSRT110B_scaff_3626_2	ADV57118.1	Ryanodine receptor 3 [*Opsariichthys bidens*]	1E-134
R50870	26	MMSRT065C_scaff_1820_1	XP_003222987.1	Cadherin-8	3E-71
R20892	28	MMSRT029H_scaff_2020_1	XP_003763770.1	Transcription factor SOX-14	4E-79
R50730	sex	MMSRT014A_scaff_2077_2	AAI63316.1	Adrenergic, alpha-2D-, receptor b	8E-173
R34426	sex	MMSRT022B_scaff_1619_1	XP_003452446.1	Sodium/calcium exchanger 1	9E-173
R03937	sex	MMSRT041G_scaff_1450_1	NP_001121839.1	Prostaglandin E2 receptor EP4 subtype	1E-100
R46658	sex	MMSRT072E_scaff_1452_1	EKC18115.1	Sperm-associated antigen 16	0

## Discussion

Migration is a central behavior in the ecology and evolution of many organisms. Both migration and dispersal have a heritable component in animals as diverse as crickets (Roff and Fairbairn 2007) and birds (Hansson *et al.* 2003; Pasinelli *et al.* 2004). In salmonids, the heritability of migratory-related traits appears to be high (*e.g.*, proportion of brood smolting in *O. mykiss*; h^2^= 0.726; Thrower *et al.* 2004 and smolt timing in Atlantic Salmon, *Salmo salar*; Páez *et al.* 2011), strongly suggesting some level of underlying genetic control. To determine the genetic basis to migration, the authors of previous studies have either taken a “bottom-up” approach (*i.e.*, investigating candidate genes for variation associated with migration) or a “top-down” approach such as by constructing genetic crosses that vary in a phenotype associated with migration. In this study, we used population genomics methods to identify loci showing unique patterns of divergence between migratory and nonmigratory ecotypes in natural populations of *O. mykiss*, supporting “top-down” approaches as a tractable way to detect loci under selection in natural populations.

By using an unassembled scaffold version of the *O. mykiss* genome, we used kernel smoothing average methods to identify regions enriched for markers that deviated from the rest of the genome. The Sashin Creek population produced a large number of markers that suggest differentiation between migrants and residents in both population genetic and association approaches. The Sashin Creek *F*_ST_ comparisons suggest loci on Omy2 and Omy7 show the greatest amount of differentiation between residents and migrants. A negative peak on Omy2 is also found in the Tajima’s *D* analysis within the Sashin Creek migrants, suggesting either the effects purifying selection or rapid population expansion after a bottleneck. The historical population size of the Sashin Creek migrants is unknown and of late the number of returning fish is decreasing (F. P. Thrower, unpublished data), suggesting negative departures from neutrality in the Sashin Creek migrants are caused by purifying selection. A reduction in the nucleotide diversity is also found in the Sashin Creek migrants on Omy2, suggesting reduced variation in this region of the genome, possibly due to purifying selection for alleles involved in anadromy.

By contrast the Little Sheep Creek samples did not show strong differentiation between the migrants and the residents on Omy2. There was a departure from neutrality in the positive direction on Omy2 (the opposite from the general trend) in the Tajima’s *D* results, suggesting positive selection or population contraction. However, it is likely that selection on this locus is not connected to migration because, first, there was no such peak in the *F*_ST_ analysis, and second, both resident and migrant samples were combined for the Little Sheep Creek Tajima’s *D* analysis. The kernel smoothing *F*_ST_ outlier analysis also suggested that Omy7 contains markers divergent between the resident and migrant populations in Sashin Creek, and like Omy2 these associations are coupled with a decrease in the Tajima’s *D* value within the Sashin Creek migrants and a reduction in nucleotide diversity. The Little Sheep Creek samples, by contrast, did not show any regions of the genome suggestive of the effects of selection (or associations between genotype and phenotype; see File S5). Several other peaks of differentiation were noted from the Sashin Creek *F*_ST_ outlier analysis, namely Omy4, Omy8, Omy12, Omy14, and Omy22. Although these peaks are lower than those associated with Omy2 and Omy7, they also exhibit a negative departure from neutrality in the Sashin Creek migrants and a neutral distribution in the Sashin Creek residents, suggesting there are loci on these chromosomes that also contribute to the genetic differentiation between migrants and residents. However, we were only able to map 1990 (25%) SNPs. It is therefore possible that we have missed regions of the genome that show signatures of selection.

Comparing our results with other studies is difficult, especially because there appear to be population-specific genetic effects associated with migration/residency. For example neither Nichols *et al.* (2008) nor Hecht *et al.* (2012a) found QTL for migratory-related traits (during the juvenile phase) on Omy2. However, Hecht *et al.* (2012a) did find QTL for shape differences on Omy7. Comparisons between our results and Hecht *et al.* (2012a) are more applicable because they used a QTL scan between markers segregating between an F_2_ cross in Sashin Creek. Of the other regions of the genome with elevated differentiation in Sashin Creek, Hecht *et al.* (2012a) found QTL on Omy4, Omy8, Omy12, and Omy14 but not Omy22. Omy12 and Omy14 were found to be “hotspot QTL regions” (*i.e.*, multiple QTL for different traits), and further support for the importance of these regions comes from QTL for growth (Wringe *et al.* 2010) and osmoregulation (Le Bras *et al.* 2011). Similarly, Nichols *et al.* (2008) found Omy5 and Omy10 being “hotspot QTL regions,” and additional studies have found loci on Omy5 to be associated with migratory related traits (O’Malley *et al.* 2007; Martinez *et al.* 2011) and timing of sexual maturity and development rate (Danzmann *et al.* 2005; Leder *et al.* 2006; Nichols *et al.* 2007; Miller *et al.* 2012). Although both Omy10 and Omy5 contain outlier *F*_ST_ loci and a largely negative Tajima’s *D* distribution in the Sashin Creek migrants, kernel smoothing suggests none of these chromosomes are enriched for such loci. However, it is important to keep in mind that only 25% of the total SNPs were matched to the *O. mykiss* genome. The relatively small number of loci confidently ordered in the genome is a prevailing issue that will be important in this and other nonmodel organisms. It is clear, not only that migration/residency is a complex trait involving many genes scattered through the genome, but also that there are striking differences in the number and location of such loci between populations. However, undoubtedly not all outlier loci are connected with the evolution of migration/residency. The separation of migrant and resident samples in the Sashin Creek dataset means other factors could also (due to different selection pressures, patterns of linkage disequilibrium or drift) cause segregation that overlaps with phenotype.

The evolution of marine and freshwater adaptation has probably been best studied in the three-spined stickleback (*Gasterosteus aculeatus*) and currently this is the only current comparative model for our study. Ecological genomic studies in sticklebacks have shown very convincingly that the evolution of multiple independent freshwater populations from a marine ancestor have involved parallel genetic changes (Colosimo *et al.* 2004; Cresko *et al.* 2004; Hohenlohe *et al.* 2010). However, the situation in freshwater rainbow trout is more complex. In this study, the population history of the two study sites is very different and demography has important implications for finding signatures of selection, but perhaps more importantly in the ability to reliably test for true associations between genotypes and phenotypes using GWAS (see Price *et al.* 2006; Nielsen *et al.* 2007). The Sashin Creek system includes barriers to migration that separate the two forms (Thrower *et al.* 2004). Although migrants from the upper watershed may migrate out to the ocean, large waterfalls prevent return migration to the upper sections of the Creek and Lake (Masuda *et al.* 2009). Similar patterns of strong differentiation (high *F*_ST_ and a greater proportion of outliers) between migrants and residents above and below barriers have been found in other populations of *O. mykiss* (Narum *et al.* 2011; Limborg *et al.* 2012; Pearse *et al.* 2009; Martinez *et al.* 2011). In such systems there could be selection for above barrier fish to not produce migrant offspring as these would be lost from the population. Over time, this could result in purging of any alleles associated with anadromy. Such a scenario could produce patterns of genome variation similar to what we found in Sashin Creek with many loci associated with migration/residency, many outlier loci from *F*_ST_ analysis, high LD, a largely negative departure from neutrality in the source population, and many private alleles being present in the migrants.

What followed in such systems can be thought of as a hard selection sweep in which alleles associated with residency were selected to a much greater frequency than the same alleles in the migrant population. Further support for selection against residents producing migrants comes from Thrower and Joyce (2004), who examined the survivability of migrant fish produced from crosses between resident fish and migrant fish. They found that migrant offspring produced by migrant fish had a greater proportion of returns than migrant offspring from resident fish, suggesting migrants are inheriting alleles that promote ocean survivability. In contrast, the Little Sheep Creek samples were collected from an area where migratory and resident life history types coexist providing greater opportunity for mating between the life history types (Berntson *et al.* 2011) as confirmed by the few loci associated with migration/residency, few *F*_ST_ outliers, low levels of LD, largely neutral Tajima’s *D* values, and low numbers of private alleles. Similar low levels of *F*_ST_ and few outliers are found in other populations of *O. mykiss* where residents and migrants co-occur (Limborg *et al.* 2012). This finding suggests either an historic selective hard sweep that has been eroded by drift and interbreeding between resident and migrant forms, or a soft sweep in which selection pressure between forms is reduced.

Although migration/residency in *O. mykiss* is undoubtedly a complex trait, the long-standing issue of how a “migration syndrome” is quantified or identified can have important implications on our synthesis of the results, especially when comparing between studies. In the study herein, all migrant fish were sampled as sexually mature adults returning to their natal streams, but the two resident populations were sampled at different time points during development. The Sashin Creek resident fish were tested for gamete expression before sampling; only fish that produced gametes were included (sexual maturity precludes migration in salmonids, discussed below). In contrast, the Little Sheep Creek residents were sampled after fish reached a minimal size (fork length greater than 160 mm). Though the migrant phenotype is fairly easily scored (by the act of migration in downstream or upstream traps, distinctive body morphology and coloration), the resident phenotype may not always be definitively determined until reaching sexual maturation. Moreover, it is possible that some of the fish classified as residents in the Little Sheep Creek fish would have subsequently migrated. Similar complications arise when comparing our results with those from previous studies (Nichols *et al.* 2008; Hecht *et al.* 2012, 2013). We sampled migrants that had successfully returned from the ocean to spawn, whereas previous studies determined phenotype at 2 years of age (the point of smoltification, which occurs in the juvenile phase). This could be crucial, as the migrant fish in this study would have experienced additional selection and mortality in the ocean whereas previous studies did not. Therefore we are essentially measuring two different traits, the genetic basis of smoltification in the juvenile stage (Nichols *et al.* 2008; Hecht *et al.* 2012, 2013) and the genetic basis of successful migration (this study).

The annotation of genome scaffolds that are linked to candidate loci for directional selection produced associations to genes connected to development rate, osmoregulatory function, and reproductive maturity (Tables 1 and 2). Our BLAST results show that growth hormone 1 (*GH1*), a gene that is involved in growth and development (Dickhoff *et al.* 1997), is on the same contig as a tag that contains a marker that is associated with migration and certainly warrants further study. There are two BLASTn and four BLASTx hits to genes involved in ion exchange such as various potassium and calcium voltage-gated channels. Presumably such genes are involved in efficient osmoregulation and could be under heavy selection pressures in migratory individuals. Olfaction is also important in the ability of returning migrants to navigate to their natal streams, and an olfactory receptor (family C) is linked to a significant SNP therein (R08544, R03392).

Intriguingly, several genes involved in sexual differentiation and maturity were linked to significant SNPs in this study: gonadotropin-subunit beta-2 (*Gthb2*), *SOX14*, and sperm-associated antigen 16. Receptors for gonadotropin have been shown to be associated with return migration timing for spawning in chum salmon (*Oncorhynchus keta*; Onuma *et al.* 2010), a trait that could be under selection in all migratory salmonids. Members of the *SOX* transcription factors have been shown to be involved in sex differentiation in *O. mykiss* (Baron *et al.* 2005) and other vertebrates. There appears to be a strong relationship between age of sexual maturity and sex, with the vast majority of sexual mature precocious fish being male in *O. mykiss* (*e.g.*, Sharpe *et al.* 2011) and other species of salmonid (*e.g.*, Larsen *et al.* 2010). This could have a direct effect on migratory behavior as smoltification delays sexual maturity (Thorpe 1994). Confirming this, the Sashin Creek samples show a strong sex bias with 76% of the migrants being female compared to 29% of the residents. Of course, BLAST approaches like this are tenuous without a reference genome. We were only able to get BLAST information for a very small proportion (12%) of the significant SNPs, however they do contain some genes with functions connected to migration and warrant further analysis.

In this study, we have demonstrated the utility of RAD tag sequencing to find and genotype thousands of SNPs to understand possible adaptive divergence between migratory and resident ecotypes in natural populations. Although combining population genomic and association approaches is a powerful way to overcome some of the limitations of associations studies, GWAS in populations where phenotype is associated with strong population structure produces problems in separating true positives from loci that diverged simply as a function of demographic processes (see File S5). More generally, studies to dissect the genetic architecture of complex traits in nonmodel organisms will continue to be limited by: (1) confounding of traits with population demography, (2) the availability of a linkage map or genome sequence without which trends within and between chromosomes cannot be examined, (3) the amount of linkage disequilibrium within populations of study, (4) potentially confounding effects of environment on an individual’s phenotype, (5) the trait itself, and (6) the genetic architecture of the trait (simple, Mendelian or complex involving epistasis). With respect to the genetic basis of migration in *O. mykiss*, it is clear that many different regions of the genome are associated with this complex trait, both from this and other studies. Some of these regions seemed to be shared between different populations of *O. mykiss*, and some seem to be unique to individual populations, but note the differences in sampling returning adults in our study *vs.* smolting juveniles in other studies. We are still unsure whether this is a phenomenon unique to *O. mykiss*, salmonids or whether it is found in many different taxa that have a genetic basis to migration and only future studies in other species will allow us to answer this question. In addition, more work needs to be done to determine which genes are involved and to fully understand the function of such genes. With the genome for *O. mykiss* soon to be completed, this will hopefully soon become a possibility.

## Supplementary Material

Supporting Information

## References

[bib1] Allendorf F. Thorgaard G., 1984 Tetraploidy and the evolution of salmonid fishes, pp. 1–46 in Evolutionary Genetics of Fishes, edited by Truner B. J. Plenum Press, New York

[bib2] Amores A. Catchen J. Ferrara A. Fontenot Q. Postlethwait J. H., 2011 Genome evolution and meiotic maps by massively parallel DNA sequencing: spotted gar, an outgroup for the teleost genome duplication. Genetics 188: 799–808 21828280 10.1534/genetics.111.127324 PMC3176089

[bib3] Antao T. Lopes A. Lopes R. J., 2008 LOSITAN: A workbench to detect molecular adaptation based on a F(st)-outlier method. Bioinformatics 9: 323 18662398 10.1186/1471-2105-9-323 PMC2515854

[bib4] Baird N. Etter P. D. Atwood T. S. Currey M. C. Shiver A. L., 2008 Rapid SNP discovery and genetic mapping using sequenced RAD markers. PLoS ONE 3: e3376 18852878 10.1371/journal.pone.0003376 PMC2557064

[bib5] Baron D. Houlgatee R. Fositer A. Guiguen Y., 2005 Large-scale temporal gene expression profiling during gonadal differentiation and early gametogenesis in rainbow trout. Biol. Reprod. 73: 959–966 16014816 10.1095/biolreprod.105.041830

[bib6] Baxter S. W. Davey J. W. Johnston J. S. Shelton A. M. Heckel D. G., 2011 Linkage mapping and comparative genomics using next-generation RAD sequencing of a non-model organism. PLoS ONE 6: e19315 21541297 10.1371/journal.pone.0019315 PMC3082572

[bib7] Beaumont M. A. Nichols R. A., 1996 Evaluating loci for use in the genetic analysis of population structure. Proc. Biol. Sci. 263: 1619–1626

[bib8] Berntson E. A. Carmichael R. W. Flesher M. W. Ward E. J. Moran P., 2011 Diminished reproductive success of steelhead from a hatchery supplementation program (Little Sheep Creek, Imnaha Basin, Oregon). Trans. Am. Fish. Soc. 140: 685–698

[bib9] Chutimanitsakun Y. Nipper R. W. Cuesta-Marcos A. Cistué L. Corey A., 2011 Construction and application for QTL analysis of a restriction site associated DNA (RAD) linkage map in barley. BMC Genomics 12: 4 21205322 10.1186/1471-2164-12-4 PMC3023751

[bib10] Colosimo P. F. Peichel C. L. Nereng K. Blackman B. K. Shaprio M. D., 2004 The genetic architecture of parallel armor plate reduction in threespine sticklebacks. PLoS Biol. 2: e109 15069472 10.1371/journal.pbio.0020109 PMC385219

[bib11] Cresko W. A. Amores A. Wilson C. Murphy J. Currey M., 2004 Parallel genetic basis for repeated evolution of armor loss in Alaskan threespine stickleback populations. Proc. Natl. Acad. Sci. USA 101: 6050–6055 15069186 10.1073/pnas.0308479101 PMC395921

[bib12] Dalziel A. C. Rogers S. M. Schulte P. M., 2009 Linking genotypes to phenotypes and fitness: how mechanistic biology can inform molecular ecology. Mol. Ecol. 18: 4997–5017 19912534 10.1111/j.1365-294X.2009.04427.x

[bib13] Danzmann R. G. Cairnet M. Davidson W. S. Ferguson M. M. Gharbi K., 2005 A comparative analysis of the rainbow trout genome with 2 other species of fish (Arctic charr and Atlantic salmon) within the tetraploid derivative Salmonidae family (sub-family: Salmoninae). Genome 48: 1037–1051 16391673 10.1139/g05-067

[bib14] Davey J. W. Hohenlohe P. A. Etter P. D. Boone J. Q. Catchen J. M., 2011 Genome-wide genetic marker discovery and genotyping using next-generation sequencing. Nat. Rev. Genet. 12: 499–510 21681211 10.1038/nrg3012

[bib15] Dickhoff W. W. Beckman B. R. Larsen D. A. Duan C. Moriyama S., 1997 The role of growth in endocrine regulation of salmon smoltification. Fish Physiol. Biochem. 17: 231–236

[bib16] Dingle H., 1991 Evolutionary genetics of animal migration. Am. Zool. 31: 253–264

[bib17] Dingle H., 2006 Animal migration: is there a common migratory syndrome? J. Ornithol. 147: 212–220

[bib18] Everett M. V. Grau E. D. Seeb J. E., 2011 Short reads and nonmodel species: exploring the complexities of next-generation sequence assembly and SNP discovery in the absence of a reference genome. Mol. Ecol. Res. 11(Suppl 1): 93–108 10.1111/j.1755-0998.2010.02969.x 21429166

[bib19] Hansson B. Bensch S. Hasselquist D., 2003 Heritability of dispersal in the greet reed warbler. Ecol. Lett. 6: 290–294

[bib20] Hecht B. C. Thrower F. P. Hale M. C. Miller M. R. Nichols K. M., 2012 The genetic architecture of migration related traits in rainbow and steelhead trout, *Oncorhynchus mykiss*. G3 (Bethesda) 2: 1113–1127 22973549 10.1534/g3.112.003137 PMC3429926

[bib21] Hecht B. C. Campbell N. R. Holeck D. E. Narum S. R., 2013 Genome-wide association reveals genetic basis for the propensity to migrate in wild populations of rainbow and steelhead trout. Mol. Ecol. 22: 3061–3076 23106605 10.1111/mec.12082 PMC3609938

[bib22] Hess J. E. Campbell N. R. Close D. A. Docker M. F. Narum S. R., 2013 Population genomics of pacific lamprey: adaptive variation in a highly dispersive species. Mol. Ecol. 22: 2898–2916 23205767 10.1111/mec.12150

[bib23] Hohenlohe P. A. Bassham S. Etter P. D. Stiffler N. Johnson E. A., 2010 Population genomics of parallel adaptation in threespine stickleback using sequenced RAD tags. PLoS Genet. 6: e1000862 20195501 10.1371/journal.pgen.1000862 PMC2829049

[bib24] Hohenlohe P. A. Amish S. J. Catchen J. M. Allendorf F. W. Luikart G., 2011 Next-generation RAD sequencing identifies thousands of SNPs for assessing hybridization between rainbow and westslope cutthroat trout. Mol. Ecol. Res. 11: 117–122 10.1111/j.1755-0998.2010.02967.x 21429168

[bib25] Ingvarsson P. K. Street N. R., 2011 Association genetics of complex traits in plants. New Phytol. 189: 909–922 21182529 10.1111/j.1469-8137.2010.03593.x

[bib26] Kofler R. Pandey R. V. Schloetterer C., 2011 PoPoolation2: identifying differentatiation between populations using sequencing of pooled DNA samples (Pool-seq). Bioinformatics 27: 3435–3436 22025480 10.1093/bioinformatics/btr589 PMC3232374

[bib27] Larsen D. A. Beckman B. R. Cooper K. A., 2010 Examining the conflict between smolting and precocious male maturation in spring (Stream-type). Chinook Salmon. Trans. Am. Fish. Soc. 139: 564–578

[bib28] Le Bras Y. Dechamp N. Krieg F. Filangi O. Guyomard R., 2011 Detection of QTL with effects on osmoregulation capacities in the rainbow trout (*Oncorhynchus mykiss*). BMC Genet. 12: 46 21569550 10.1186/1471-2156-12-46 PMC3120726

[bib29] Leder E. H. Danzmann R. G. Ferguson M. M., 2006 The candidate gene, Clock, localizes to a strong spawning time quantitative trait locus region in rainbow trout. J. Hered. 97: 74–80 16407529 10.1093/jhered/esj004

[bib30] Lewontin R. C., 1964 The interaction of selection and linkage. I. General considerations; heterotic models. Genetics 49: 49–67 17248194 10.1093/genetics/49.1.49 PMC1210557

[bib31] Liedvogel M. Akesson S. Bensch S., 2011 The genetics of migration on the move. Trends Ecol. Evol. 26: 561–569 21862171 10.1016/j.tree.2011.07.009

[bib32] Limborg M. T. Blankenship S. M. Young S. F. Utter F. M. Seeb L. W., 2012 Signatures of natural selection among lineages and habitats in *Oncorhynchus mykiss*. Ecol. Evol. 2: 1–18 22408722 10.1002/ece3.59 PMC3297173

[bib33] Luikart G. England P. R. Tallmon D. Jordan S. Taberlet P., 2003 The power and promise of population geneomics: from genoytping to genome typing. Nat. Rev. Genet. 4: 981–994 14631358 10.1038/nrg1226

[bib34] Martínez A. Garza J. C. Pearse D. E., 2011 A microsatellite genome screen identifies chromosomal regions under differential selection in steelhead and rainbow trout. Trans. Am. Fish. Soc. 140: 829–842

[bib35] Masuda M. Thrower F. P. Nichols K. M., 2009 The effects of violating Hardy – Weinberg equilibrium assumptions on a cluster- based population mixture analysis of steelhead populations in Southeast Alaska. N. Am. J. Fish. Manage. 29: 37–41

[bib36] Miller M. R. Dunham J. P. Amores A. Cresko W. A. Johnson E. A., 2007 Rapid and cost-effective polymorphism identification and genotyping using restriction site associated DNA (RAD) markers. Genome Res. 17: 240–248 17189378 10.1101/gr.5681207 PMC1781356

[bib37] Miller M. R. Brunelli J. P. Wheeler P. A. Liu S. Rexroad C. E., 2012 A conserved haplotype controls parallel adaptation in geographically distant salmonid populations. Mol. Ecol. 21: 237–249 21988725 10.1111/j.1365-294X.2011.05305.x PMC3664428

[bib38] Narum S. R. Zendt J. S. Frederiksen C. Campbell N. Matala A., 2011 Candidate genetic markers associated with anadromy in *Oncorhynchus mykiss* of the Klickitat river. Trans. Am. Fish. Soc. 140: 843–854

[bib39] Nielsen R., 2005 Molecular signatures of natural selection. Annu. Rev. Genet. 39: 197–218 16285858 10.1146/annurev.genet.39.073003.112420

[bib40] Nielsen R. Hellmann I. Hubisz M. Bustamante C. Clark A. G., 2007 Recent and ongoing selection in the human genome. Nat. Rev. Genet. 8: 857–868 17943193 10.1038/nrg2187 PMC2933187

[bib41] Nielsen R. Hubisz M. J. Hellmann I. Torgerson D. Andres A. M., 2009 Darwinian and demographic forces affecting human protein coding genes. Genome Res. 19: 838–849 19279335 10.1101/gr.088336.108 PMC2675972

[bib42] Nichols K. M. Broman K. W. Sundin K. Young J. M. Wheeler P. A., 2007 Quantitative trait loci x maternal cytoplasmic environment interaction for development rate in *Oncorhynchus mykiss*. Genetics 175: 335–347 17057232 10.1534/genetics.106.064311 PMC1774986

[bib43] Nichols K. M. Edo A. F. Wheeler P. A. Thorgaard G. H., 2008 The genetic basis of smoltification-related traits in *Oncorhynchus mykiss*. Genetics 179: 1559–1575 18562654 10.1534/genetics.107.084251 PMC2475755

[bib44] Norman J. D. Danzmann R. G. Glebe B. Ferguson M. M., 2011 The genetic basis of salinity tolerance traits in Arctic charr (*Salvelinus alpinus*). BMC Genet. 12: 81 21936917 10.1186/1471-2156-12-81 PMC3190344

[bib45] O’Malley K. G. Camara M. D. Banks M. A., 2007 Candidate loci reveal genetic differentiation between temporally divergent migratory runs of Chinook salmon (*Oncorhynchus tshawytscha*). Mol. Ecol. 16: 4930–4941 17971087 10.1111/j.1365-294X.2007.03565.x

[bib46] Onuma T. A. Ban M. Makino K. Katsumata H. Hu W., 2010 Changes in gene expression for GH/PRL/SL family hormones in the pituitaries of homing chum salmon during ocean migration through upstream migration. Gen. Comp. Endocrinol. 166: 537–548 20100485 10.1016/j.ygcen.2010.01.015

[bib47] Páez D. J. Brisson-bonenfant C. Rossignol O. Guderley H. E. Bernatchez L., 2011 Alternative developmental pathwats and the propensity to migrate: a case study in the Atlantic salmon. J. Evol. Biol. 24: 245–255 21044203 10.1111/j.1420-9101.2010.02159.x

[bib48] Palti Y. Genet C. Luo M. C. Charlet A. Gao G. T., 2011 A first generation integrated map of the rainbow trout genome. BMC Genomics 12: 180 21473775 10.1186/1471-2164-12-180 PMC3079668

[bib49] Pasinelli G. Schiegg K. Walters J. R., 2004 Genetic and environmental influences on natal dispersal distance in a resident bird species. Am. Nat. 164: 660–669 15540155 10.1086/424765

[bib50] Peakall R. Smouse P. E., 2006 GENALEX 6: genetic analysis in excel. Population genetic software for teaching and research. Mol. Ecol. Notes 6: 288–295 10.1093/bioinformatics/bts460 PMC3463245 22820204

[bib51] Pearse D. E. Hayes S. A. Bond M. H. Hanson C. V. Anderson E. C., 2009 Over the falls? Rapid evolution of ecotypic differentiation in steelhead/rainbow trout (*Oncorhynchus mykiss*). J. Hered. 100: 515–525 19561050 10.1093/jhered/esp040

[bib52] Pfender W. F. Saha M. C. Johnson E. A. Slabaugh M. B., 2011 Mapping with RAD (restriction-site associated DNA) markers to rapidly identify QTL for stem rust resistance in *Lolium* perenne. Theor. Appl. Genet. 122: 1467–1480 21344184 10.1007/s00122-011-1546-3

[bib53] Phillips R. B. Nichols K. M. De Koning J. J. Morasch M. R. Keatley K. A., 2006 Assignment of rainbow trout linkage groups to specific chromosomes. Genetics 174: 1661–1670 16951085 10.1534/genetics.105.055269 PMC1667062

[bib54] Price A. L. Patterson N. J. Plenge R. M. Weinblatt M. E. Shadick N. A., 2006 Principal components analysis corrects for stratification in genome-wide association studies. Nat. Genet. 38: 904–909 16862161 10.1038/ng1847

[bib55] Pulido F. Berthold P., 2003 Quantitative Genetic Analysis of Migratory Behaviour. Springer-Verlag Berlin, Berlin

[bib56] Roff D. A. Fairbairn D. A., 2007 The evolution and genetics of migration in insects. Bioscience 57: 155–164

[bib57] Rowe H. C. Renaut S. Guggisberg A., 2011 RAD in the realm of next-generation sequencing technologies. Mol. Ecol. 20: 3499–3502 21991593 10.1111/j.1365-294x.2011.05197.x

[bib58] Seeb J. E. Carvalho G. Hauser L. Naish K. Roberts S., 2011 Single-nucleotide polymorphism (SNP) discovery and applications of SNP genotyping in nonmodel organisms. Mol. Ecol. Res. 11: 1–8 10.1111/j.1755-0998.2010.02979.x 21429158

[bib59] Sharpe C. S. Beckman B. R. Cooper K. A. Hulett P. L., 2011 Growth modulation during juvenile rearing can reduce rates of residualism in the progeny of wild steelhead broodstock. J. Fish. Manag. 27: 37–41

[bib60] Tajima F., 1989 Statistical method for testing the neutral mutation hypothesis by DNA polymorphism. Genetics 123: 585–595 2513255 10.1093/genetics/123.3.585 PMC1203831

[bib61] Thorpe J. E., 1994 An alternative view of smolting in salmonids. Aquaculture 121: 105–113

[bib62] Thrower F. P. Joyce J. E., 2004 Effects of 70 years of freshwater residency on survival, growth, early maturation and smolting in a stock of anadromous rainbow trout from southeast Alaska. Am. Fish. Soc. Symp. 44: 485–496

[bib63] Thrower F. P. Hard J. J. Joyce J. E., 2004 Genetic architecture of growth and early life-history transitions in anadromous and derived freshwater populations of steelhead. J. Fish Biol. 65: 286–307

[bib64] Wasko A. P. Martins C. Oliveira C. Foresti F., 2003 Non-destructive genetic sampling in fish. An improved method for DNA extraction from fish fins and scales. Hereditas 138: 161–165 14641478 10.1034/j.1601-5223.2003.01503.x

[bib70] Watterson G. A., 1975 On the number of segregating sites in genetical models without recombination. Theoret. Popul. Biol. 7: 256–276 1145509 10.1016/0040-5809(75)90020-9

[bib65] Wilcove D. S. Wikelski M., 2009 Going, going, gone: is animal migration disappearing. PLoS Biol. 6: e188 18666834 10.1371/journal.pbio.0060188 PMC2486312

[bib66] Wringe B. F. Devlin R. H. Ferguson M. M. Moghadam H. K. Sakhrani D., 2010 Growth-related quantitative trait loci in domestic and wild rainbow trout (*Oncorhynchus mykiss*). BMC Genet. 11: 63 20609225 10.1186/1471-2156-11-63 PMC2914766

