# Supplementary material for: Evaluating Adaptive Divergence Between Migratory and Nonmigratory Ecotypes of a Salmonid Fish, Oncorhynchus mykiss
Source: G3 (Bethesda). 2013 Aug 1;3(8):1273–85. doi: 10.1534/g3.113.006817 (PMC3737167; doi:10.1534/g3.113.006817)
Supplement: Supporting Information [file supp_3_8_1273__index.html]

Evaluating Adaptive Divergence Between Migratory and Nonmigratory Ecotypes of a Salmonid Fish, Oncorhynchus mykiss — Supporting Information 

# Evaluating Adaptive Divergence Between Migratory and Nonmigratory Ecotypes of a Salmonid Fish, *Oncorhynchus mykiss*

## Supporting Information for Hale *et al.*, 2013

**Files in this Data Supplement:**

- Supporting Information - Files S1-S5 (PDF, 1 MB)
- File S1 - Reads per individual from Illumina RADseq (PDF, 61 KB)
- File S5 - Details of GWAS methods and results (PDF, 1 MB)
- File S2 - Name and sequence of SNPs that did not match Miller *et al.* (2012). (.xls, 4 MB)
- File S3 - Names and statistics of loci with a significant population genomic statistic (.xls, 249 KB)
- File S4 - Sequences of 61 scaffolds (Miller *et al.* pers. comm.) that produced both a significant association in at least one of the population genomic and/or GWAS tests and an annotation from either BLASTn or BLASTx analysis. (.txt, 3 MB)
