# Supplementary material for: Evaluating Adaptive Divergence Between Migratory and Nonmigratory Ecotypes of a Salmonid Fish, Oncorhynchus mykiss
Source: G3 (Bethesda). 2013 Aug 1;3(8):1273–85. doi: 10.1534/g3.113.006817 (PMC3737167; doi:10.1534/g3.113.006817)

G3 Genes | Genomes | Genetics

Evaluating adaptive divergence between migratory and non-migratory ecotypes of a salmonid fish, *Oncorhynchus mykiss*

Matthew C. Hale[*], Frank P. Thrower[†], Ewann A. Berntson[‡], Michael R. Miller[§] and Krista M. Nichols[*,‡,1]

[*]Department of Biological Sciences, Purdue University, West Lafayette, Indiana
[†]National Oceanic and Atmospheric Administration, National Marine Fisheries Service, Alaska Fisheries Science Center, Ted Stevens Marine Institute, Juneau, Alaska
[‡]National Oceanic and Atmospheric Administration, National Marine Fisheries Service, Northwest Fisheries Science Center, Seattle, Washington
[§]Institute of Molecular Biology, University of Oregon, Eugene, Oregon

[1]Corresponding author

DOI: 10.1534/g3.113.006817

File S1

Reads per individual from Illumina RADseq

Sequencing statistics for Illumina RADseq. Columns include the sequencing chemistry (both Illumina HiSeq and HiScanSeq were used), the number of individuals pooled per library, the raw number of reads (in millions) the number of quality filtered targeted reads (in millions) and the average number of quality filtered reads per individual (in millions).

library name	chemistry	raw number of reads (million reads)	number of individuals	target QF sequences (million reads)	average QF sequences per indidivual (million reads)
LSC_SH_4	HiScan	82.4	15	54.8	3.55
LSC_RT_5	HiScan	78	15	43.3	2.89
LSC_RT_6	HiScan	78	15	39.7	2.65
SAS_SH_3	HiScan	99.4	15	15.9	10.70
LSC_RT_3	HiSeq	204.9	20	45.3	2.27
LSC_SH_3	HiSeq	205.4	15	18.9	1.26
SAS_SH_3	HiSeq	82.2	15	24.4	1.60
SAS_RT_3	HiSeq	132	15	58	3.87
SAS_Sh2	HiSeq	89.7	10	70.7	7.10
LSC_SH2	HiSeq	90	10	74.5	7.40
SAS_SH8	HiScan	77.8	15	45.1	3.00
SAS_SH9	HiScan	73.4	15	47.2	3.10
SAS_SH10	HiScan	89.5	15	51.9	3.50
LSC_SH6	HiScan	75.7	15	23.6	1.60
LSC_SH7	HiScan	84.7	15	27.8	1.90
LSC_SH8	HiScan	78.9	15	44.9	3.00
LSC_RT7	HiScan	86.8	15	41	2.70
LSC_RT11	HiScan	88.4	15	56.3	3.75
lsc_rt12	HiScan	58.3	15	39.3	2.62
lsc_rt9	HiScan	81.9	15	44.9	2.99
lsc_sh11	HiScan	83.4	15	37.9	2.53
lsc_sh12	HiScan	76.1	15	33.1	2.21
SAS_RT11	HiScan	80.1	15	51.1	3.41
SAS_RT12	HiScan	35.7	15	19.6	1.31
sas_sh11	HiScan	68.1	15	12.03	0.80
sas_sh12	HiScan	69.1	15	20.6	1.37

Files S2-S4

Available for download at http://www.g3journal.org/lookup/suppl/doi:10.1534/g3.113.006817/-/DC1

File S2 Name and sequence of SNPs that did not match Miller *et al*. (2012). Naming follows on from the database used in Miller *et al* (2012) and sequences for both allele 1 and allele 2 are given.

File S3 Names and statistics of loci with a significant population genomic statistic.

File S4 Sequences of 61 scaffolds (Miller *et al* pers. comm.) that produced both a significant association in at least one of the population genomic and/or GWAS tests and an annotation from either BLASTn or BLASTx analysis**.**

File S5

Details of GWAS methods and results

GWAS of migration/residency in two populations of *O. mykiss*

Overview

Though population structure is completely confounded with phenotype in the Sashin Creek system, we opted to conduct a GWAS to ask the following questions: 1) how severe is the confounding, and what happens when we control for population structure using 'neutral loci' identified from population genomics approaches? and 2) since population structure is not confounded with phenotype in Little Sheep Creek, what loci are associated with the phenotype when controlling for population structure in the same way?

Methods

A mixed model approach was used to evaluate associations between genotype and phenotype in each of the three datasets: Sashin only, Little Sheep Creek only, and the combined data set. This model included sex and population structure as co-factors, and an FDR correction (alpha =0.05; BENJAMINI and YEKUTIELI 2001) was applied to account for multiple testing. To account for population structure, a principal component analysis using genotypes from all neutral markers from the output from LOSITAN on the combined dataset was performed. Principle component analysis has been shown to accurately account for structure whilst being computationally faster than using, for example, STRUCTURE (PRICE *et al.* 2006; ZHAO *et al.* 2007). Principle component analysis with the 3,801 neutral loci identified from LOSITAN from the combined dataset identified three groupings of samples: the Little Sheep Creek individuals (composed of both residents and migrants), the residents from Sashin Creek and the migrants from Sashin Creek (Figure S1). The first three axes account for 12.6, 3.3 and 2.8 % of the variation respectively and were used as fixed effects in GWAS models.

To test the effectiveness of the mixed models in removing false positives, quantile-quantile plots were constructed.

Results and Discussion

Mixed models were used for GWAS on each of the three datasets separately for models for two different traits: 1) migration coded as a binary trait (0 = migrants, 1 = residents) and 2) fork length of the fish. Population structure was included as a fixed effect. Sex was also used as a fixed effect since migration has a well-known sex bias with females being more likely to migrate than males (DELLEFORS and FAREMO 1988; JONSSON *et al.* 1998). A total of 295 SNPs (p = <0.001) were significantly associated with migration in the Sashin Creek population. The strongest association between SNP genotype and migration in Sashin Creek was with SNP R41941 (FDR corrected p = 1.406×10^{-5}; Table S1). For the majority of significant associations the migrant phenotypes showed a more even distribution of genotypes between the three genotypic classes (but note that heterozygote deficits occur in several SNPs) than the resident phenotype (Table S1). The Sashin Creek fork length GWAS produced six significant SNPs. SNP R51797 produced the most significant association (FDR corrected p = 0.002 Table S2). The binary trait GWAS in Little Sheep Creek samples failed to find any statistically significant SNPs (data not shown). The fork length GWAS for Little Sheep Creek produced eight significant associations. The most significant association was with SNP 49559 (FDR corrected p = 0.001 Table S2). The combined binary GWAS failed to produce and significant associations, whereas the combined fork length GWAS produced 9 significant associations with the strongest being with SNP R50174 (FDR corrected p = 7.53×10^{-4}). No loci were significant in both the binary and fork length GWAS within the Sashin Creek populations. Very few significant loci were in common between the fork length GWAS analyses in the three data sets. Only R41941 was significant in both the Sashin Creek and combined datasets and only R50490 was significant in both the Little Sheep Creek and combined datasets. Mapping data showed every chromosome except Omy27 had at least one significant SNP for the binary GWAS in the Sashin Creek dataset (Figure S2a). Kernel smoothing suggests a relatively constant distribution of associations with no obvious chromosome containing a peak. However Omy6 and Omy16 seem to produce a slightly higher peak than the other chromosomes and Omy2 Omy7 and Omy10 also showing a slight increase the kernel-smoothed average p value. The Sashin Creek fork length GWAS produced seven mapped loci that were significant (FDR corrected p=< 0.05) which were found on Omy4, Omy5, Omy10, Omy12, Omy17 and Omy21 (Figure S2b). Kernel smoothing methods showed no chromosomes with a notable peak of associations. The Little Sheep Creek fork length GWAS produced 13 mapped loci that were significant, (FDR corrected p = < 0.05) which were located on Omy1, Omy3, Omy4, Omy5, Omy7, Omy10, Omy16, and Omy26. Kernel smoothing methods showed no chromosomes with any

peaks of association (Figure S2c). Quantile-quantile plots were constructed to test for effectiveness of removing potential false positives (due to confounding with population structure) from the model. The Sashin Creek binary GWAS shows that even after correcting for population structure there is still inflation of p-values and therefore the presence of potential false positives. However, both fork length GWAS for Sashin Creek and Little Sheep Creek suggest over correction of p-values and therefore potential false negatives (Figure S3).

Comparing population genomic and association approaches. Of the 317 loci significantly associated with either the binary migratory trait or fork length, 61 were also suggestive of positive selection in *FST* outlier analysis in at least one population. A total of 55 out of 295 loci were significant in both the outlier and associations analyses within the Sashin Creek population, the remaining 6 where significant in both the Little Sheep Creek fork length GWAS and were outliers in the Sashin Creek *FST* analysis.

Table S1 List of 20 most significant SNPs from GWAS (binary) between resident and migrants for Sashin Creek. All p-values are FDR adjusted (alpha = 0.05). The number of individuals for each of the three genotypes is also given.

		migrants			residents		
SNP ID	$-\log_{10} P$	1,1	1,2	2,2	1,1	1,2	2,2
R41941	4.852	14	10	35	65	17	7
R50379	4.852	54	6	8	11	30	47
R19982	4.852	80	1	1	16	43	28
R41412	4.852	59	6	5	7	40	41
R50823	4.852	71	4	1	8	47	33
R07776	4.852	36	9	15	6	21	60
R23382	4.852	39	10	15	4	24	60
R34439	4.852	6	6	53	53	27	7
R49259	4.852	7	4	52	36	42	6
R03501	4.833	61	8	6	8	34	42
R23606	4.833	64	3	5	13	39	34
R22532	4.814	58	2	5	12	34	39
R42248	4.809	67	3	1	16	42	29
R25897	4.697	45	11	5	7	37	40
R48260	4.417	52	6	5	11	40	37
R23109	4.332	50	6	5	21	38	24
R07673	4.332	33	16	22	8	42	35
R15784	4.332	31	21	33	19	41	26
R31269	4.332	40	24	24	11	45	26
R49942	4.332	45	11	11	22	45	19

Table S2 SNPs significant for GWAS on fork length for Sashin Creek samples (above the line) and Little Sheep Creek samples (below the line) P values are adjusted with an FDR with alpha = 0.05 (according to Benjamini and Yekutieli 2001). The number of individuals for each of the three genotypes is also given.

		migrants			residents		
SNP ID	$-\log_{10} P$	1,1	1,2	2,2	1,1	1,2	2,2
R51797	2.698	0	1	48	0	0	65
R50440	2.384	40	10	19	25	37	18
R46277	2.214	31	5	13	49	36	2
R49907	1.965	0	3	58	0	0	87
R50225	1.727	0	6	43	0	0	47
R49285	1.313	0	4	48	0	5	60
R09793	1.296	60	2	2	84	0	0
R49559	2.060	0	4	54	0	26	36
R21331	2.060	0	26	45	0	5	68
R36256	2.060	24	17	7	7	18	25
R52063	1.674	28	22	11	10	16	28
R50490	1.490	72	0	0	72	15	0
R32318	1.490	0	31	33	0	13	65
R60354	1.490	0	11	74	0	0	99

Figure S1 Two axes from a principal component analysis on: a) Sashin Creek samples, b) Little Sheep Creek samples and c) all samples.

A. First two axes from Principle Component Analysis on all 10921 SNPs used for GWAS analysis on fish from Sashin Creek, b) Little sheep creek and c) all samples. Steelhead samples are depicted by filled circles, rainbows by open circles. Only the first two axes were found to be significantly associated with the genotypes (axis 1 p = <0.001, axis 2 p = 0.04) as determined by ANOVA tests.

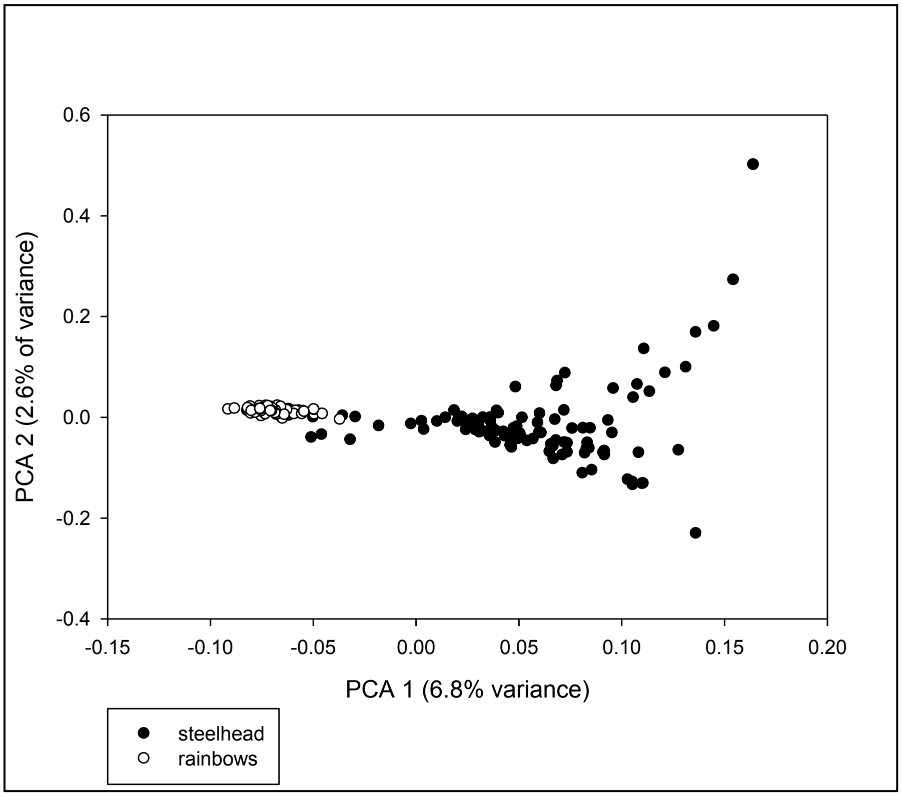

B

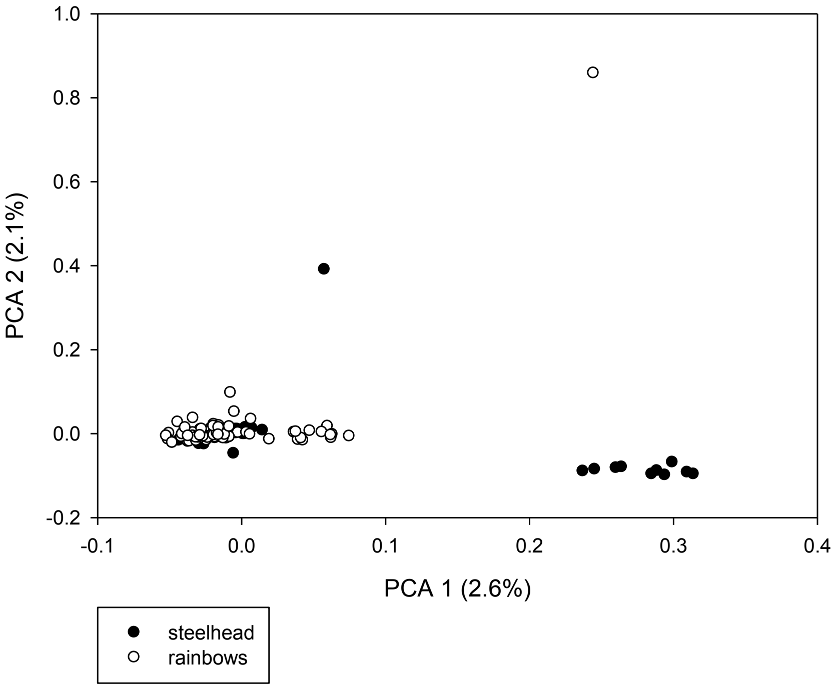
1.0
0.8
0.6
0.4
0.2
0.0
-0.2
PCA 2 (2.1%)
-0.1
0.0
0.1
0.2
0.3
0.4
PCA 1 (2.6%)
steelhead
rainbows

C

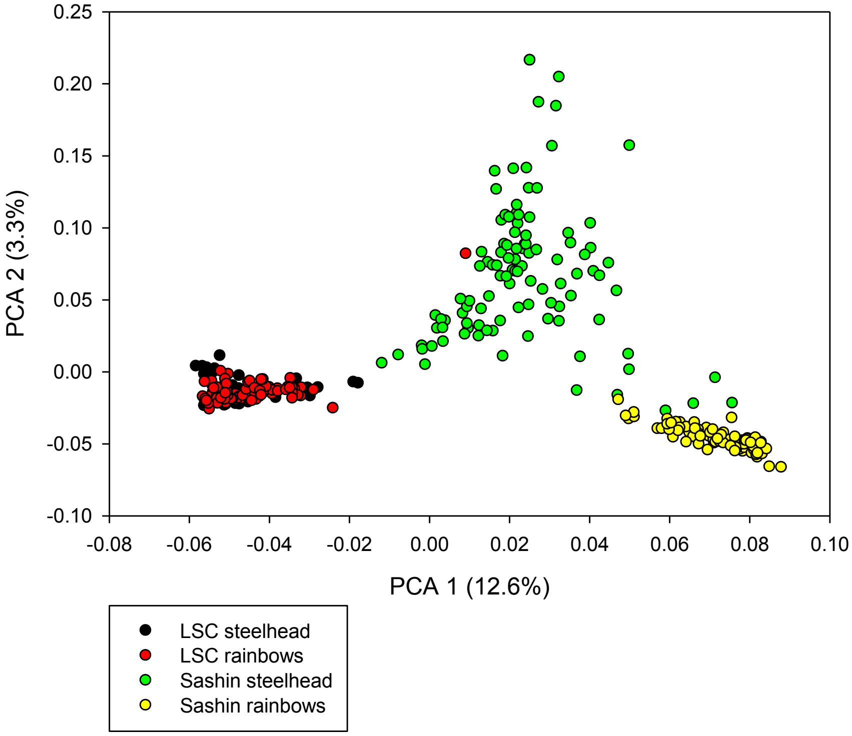
0.25
0.20
0.15
0.10
0.05
0.00
-0.05
-0.10
PCA 2 (3.3%)
-0.08
-0.06
-0.04
-0.02
0.00
0.02
0.04
0.06
0.08
0.10
PCA 1 (12.6%)
LSC steelhead
LSC rainbows
Sashin steelhead
Sashin rainbows

Figure S2 GWAS results for markers that mapped to two linkage maps of the *O. mykiss* genome (Miller *et al*. 2012; Hecht *et al.* 2012 a). The red line marks significance with an FDR correction of 0.001 and the green line significance with an FDR corrected P of 0.05. The kernel smoothed average is used for all markers and chromosomes are alternatively shaded. Plots represent GWAS results from A) Sashin Creek binary, B) Sashin Creek fork length and C) Little Sheep Creek fork length.

A

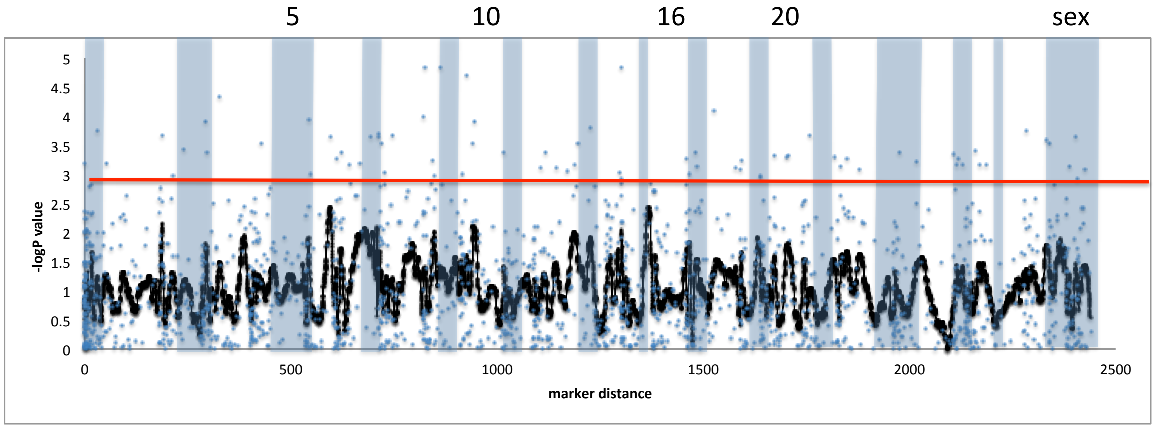

B

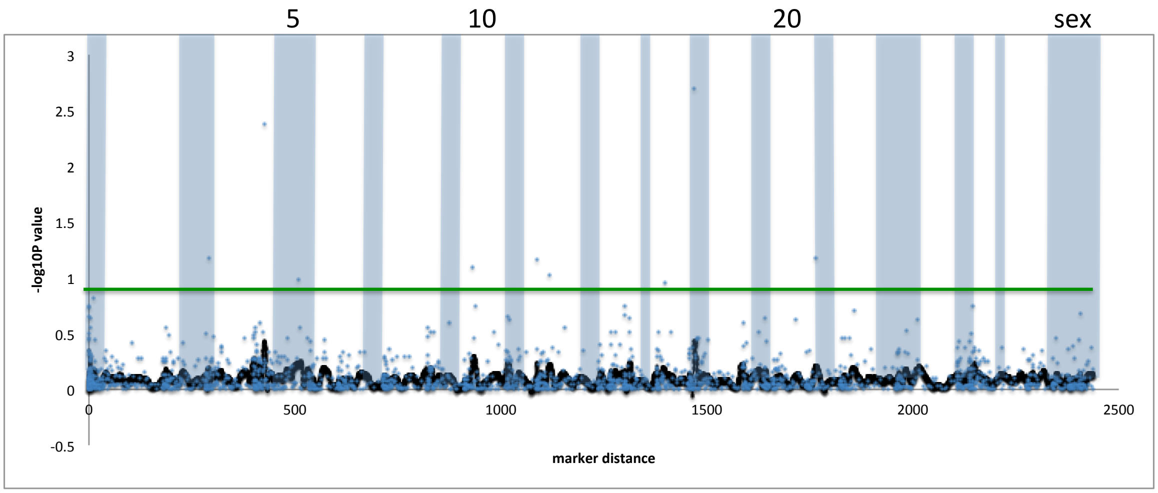

c

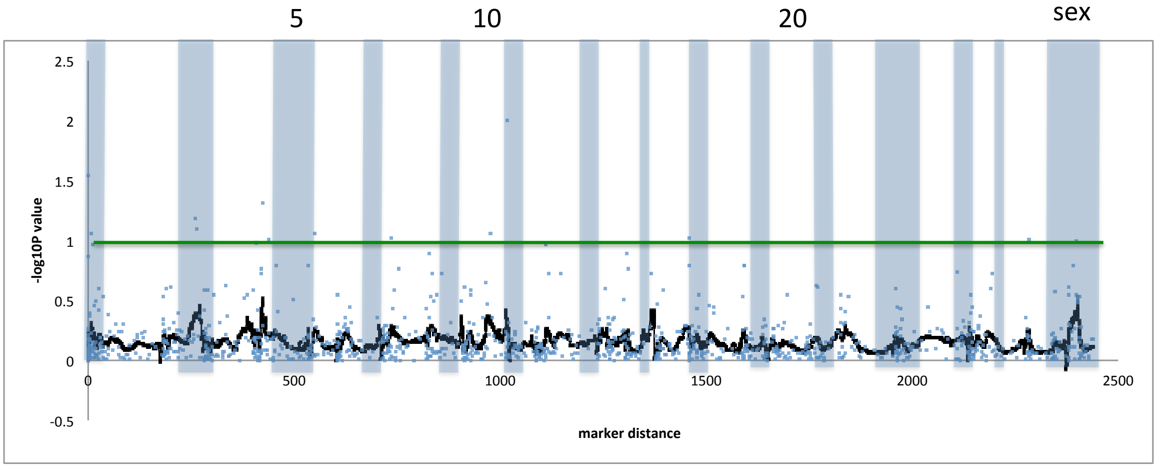
5
10
20
sex
2.5
2
1.5
1
0.5
0
-0.5
-log10P value
0
500
1000
1500
2000
2500
marker distance

Figure S3 Q-Q plots for each of the GWAS models performed a) Sashin Creek binary GWAS, b) Sashin Creek fork length GWAS, c) Little Sheep Creek GWAS.

A

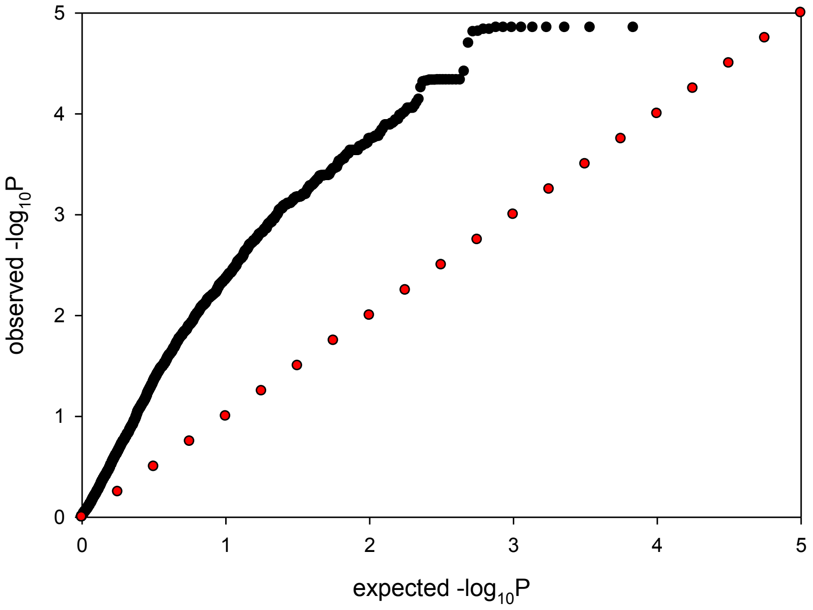

B

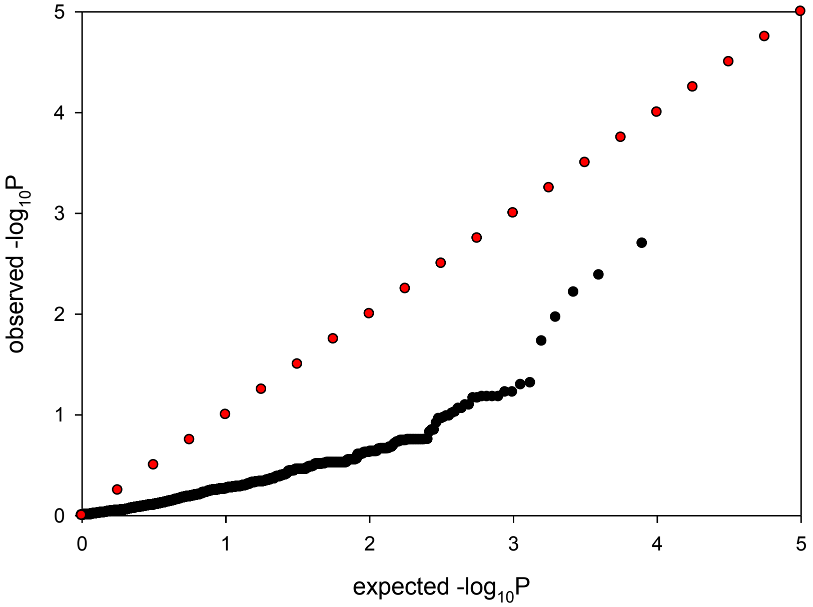

C

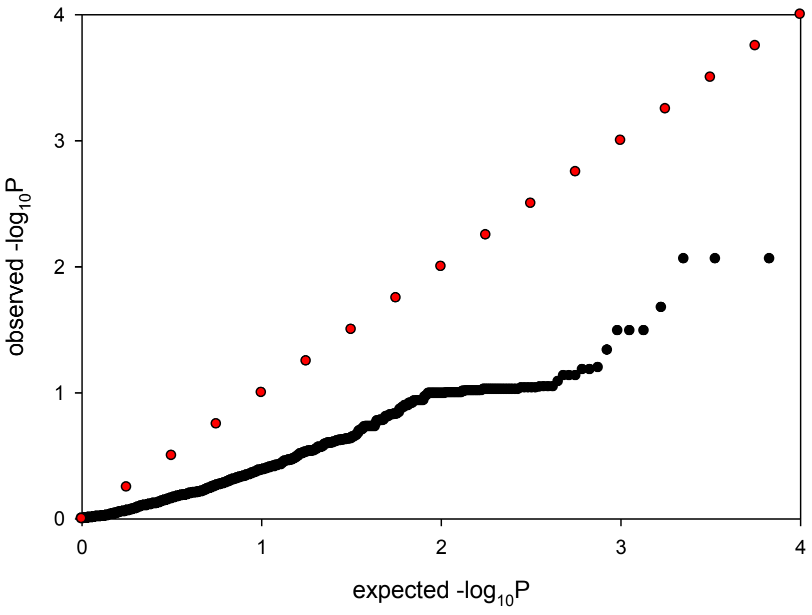

Supplement: Supporting Information [file supp_g3.113.006817_006817SI.pdf]
