# Supplementary material for: Evaluating Adaptive Divergence Between Migratory and Nonmigratory Ecotypes of a Salmonid Fish, Oncorhynchus mykiss
Source: G3 (Bethesda). 2013 Aug 1;3(8):1273–85. doi: 10.1534/g3.113.006817 (PMC3737167; doi:10.1534/g3.113.006817)
Supplement: Supporting Information [file supp_g3.113.006817_FileS1.pdf]

File S1

Reads per individual from Illumina RADseq

Sequencing statistics for Illumina RADseq. Columns include the sequencing chemistry (both Illumina HiSeq and HiScanSeq were used), the number of individuals pooled per library, the raw number of reads (in millions) the number of quality filtered targeted reads (in millions) and the average number of quality filtered reads per individual (in millions).

library name	chemistry	raw number of reads (million reads)	number of individuals	target QF sequences (million reads)	average QF sequences per indidivual (million reads)
LSC_SH_4	HiScan	82.4	15	54.8	3.55
LSC_RT_5	HiScan	78	15	43.3	2.89
LSC_RT_6	HiScan	78	15	39.7	2.65
SAS_SH_3	HiScan	99.4	15	15.9	10.70
LSC_RT_3	HiSeq	204.9	20	45.3	2.27
LSC_SH_3	HiSeq	205.4	15	18.9	1.26
SAS_SH_3	HiSeq	82.2	15	24.4	1.60
SAS_RT_3	HiSeq	132	15	58	3.87
SAS_Sh2	HiSeq	89.7	10	70.7	7.10
LSC_SH2	HiSeq	90	10	74.5	7.40
SAS_SH8	HiScan	77.8	15	45.1	3.00
SAS_SH9	HiScan	73.4	15	47.2	3.10
SAS_SH10	HiScan	89.5	15	51.9	3.50
LSC_SH6	HiScan	75.7	15	23.6	1.60
LSC_SH7	HiScan	84.7	15	27.8	1.90
LSC_SH8	HiScan	78.9	15	44.9	3.00
LSC_RT7	HiScan	86.8	15	41	2.70
LSC_RT11	HiScan	88.4	15	56.3	3.75
lsc_rt12	HiScan	58.3	15	39.3	2.62
lsc_rt9	HiScan	81.9	15	44.9	2.99
lsc_sh11	HiScan	83.4	15	37.9	2.53
lsc_sh12	HiScan	76.1	15	33.1	2.21
SAS_RT11	HiScan	80.1	15	51.1	3.41
SAS_RT12	HiScan	35.7	15	19.6	1.31
sas_sh11	HiScan	68.1	15	12.03	0.80
sas_sh12	HiScan	69.1	15	20.6	1.37
